# Plant growth-promoting rhizobacteria: Salt stress alleviators to improve crop productivity for sustainable agriculture development

**DOI:** 10.3389/fpls.2022.1101862

**Published:** 2023-01-12

**Authors:** Kailash Chand Kumawat, Barkha Sharma, Sharon Nagpal, Ajay Kumar, Shalini Tiwari, Ramakrishnan Madhavan Nair

**Affiliations:** ^1^ Department of Industrial Microbiology, Jacob Institute of Biotechnology and Bioengineering, Sam Higginbottom University of Agriculture, Technology and Sciences (SHUATS), Prayagraj, Uttar Pradesh, India; ^2^ Department of Microbiology, G. B. Pant University of Agriculture & Technology, Pantnagar, Uttarakhand, India; ^3^ Department of Microbiology, Punjab Agricultural University, Ludhiana, Punjab, India; ^4^ World Vegetable Centre, International Crops Research Institute for the Semi-Arid Tropics (ICRISAT), Patancheru, India

**Keywords:** climate stress, CRISPR, genomics, plant growth promoting rhizobacteria, proteomics

## Abstract

Soil salinity, a growing issue worldwide, is a detrimental consequence of the ever-changing climate, which has highlighted and worsened the conditions associated with damaged soil quality, reduced agricultural production, and decreasing land areas, thus resulting in an unsteady national economy. In this review, halo-tolerant plant growth-promoting rhizo-microbiomes (PGPRs) are evaluated in the salinity-affected agriculture as they serve as excellent agents in controlling various biotic–abiotic stresses and help in the augmentation of crop productivity. Integrated efforts of these effective microbes lighten the load of agro-chemicals on the environment while managing nutrient availability. PGPR-assisted modern agriculture practices have emerged as a green strategy to benefit sustainable farming without compromising the crop yield under salinity as well as salinity-affected supplementary stresses including increased temperature, drought, salinity, and potential invasive plant pathogenicity. PGPRs as bio-inoculants impart induced systemic tolerance (IST) to plants by the production of volatile organic compounds (VOCs), antioxidants, osmolytes, extracellular polymeric substances (EPS), phytohormones, and ACC-deaminase and recuperation of nutritional status and ionic homeostasis. Regulation of PGPR-induced signaling pathways such as MAPK and CDPK assists in salinity stress alleviation. The “Next Gen Agriculture” consists of the application of designer crop microbiomes through gene editing tools, for instance, CRISPR, and engineering of the metabolic pathways of the microbes so as to gain maximum plant resistance. The utilization of omics technologies over the traditional approaches can fulfill the criteria required to increase crop yields in a sustainable manner for feeding the burgeoning population and augment plant adaptability under climate change conditions, ultimately leading to improved vitality. Furthermore, constraints such as the crop specificity issue of PGPR, lack of acceptance by farmers, and legal regulatory aspects have been acknowledged while also discussing the future trends for product commercialization with the view of the changing climate.

## 1 Introduction

Soil salinity exerts severe pressure on agricultural productivity, and the devastating consequence of salt deposition in agricultural soils has escalated into a major environmental hazard. Attributed to climate change, major cereal crops have suffered yield losses with reductions of approximately 3.8% and 5.5% for maize and wheat, respectively (Lobell et al., 2011; Lipper et al., 2014). The changing climatic conditions are followed by major increases in worldwide temperature and the emergence of additional abiotic stresses, which is detrimental for agricultural output. Consequently, microbial inoculation may provide naturally derived solutions to changing climatic conditions by modulating crop development and several associated agroecosystem activities and services (Eggermont et al., 2015). The rhizosphere, rhizoplane, and endosphere are the regions of highest microbial activity where rhizosphere represents the soil available area in the vicinity of the roots, rhizoplane is the root surface, and endosphere is the spaces between plant cells (Reinhold-Hurek et al., 2015). These microbiomes have a considerable role in the modulation of crop growth and agriculture productivity along with the carbon sequestration and efficiency of phyto-remediation (Ojuederie and Babalola, 2017; Berlanas et al., 2019). Soil microbial diversity is the backbone of agroecosystem productivity, and recently, the exploration of the contribution of PGPR has made them a vital and attractive resource for sustainable agriculture. Notably, the constitution of the bacterial population in the whole ecosystem is strongly associated with the nature of the soil, which can be the reason why microbial populations can regulate greenhouse gas (GHG) emission (Ho et al., 2017). Meanwhile, rhizobacteria also facilitate active carbon cycling between the pedosphere and atmosphere along with reducing soil carbon depletion *via* their metabolism (Bardgett et al., 2008).

Rhizo-microbial diversity and abundance are greatly influenced by host plants, soil characteristics, and other environmental factors (Qiao et al., 2017). Interestingly, reports have confirmed that the beneficial microbiota of plants not only promote crop yields but also reinforce the biotic and abiotic stress tolerance activity of crops (Backer et al., 2018; Lyu et al., 2020). In general, there are three predominant pathways that microbial inoculants can influence agricultural production related to climate change: directly with the inoculants having specialized roles related to climate change; indirectly through altered plant growth and development; and indirectly through the alteration of the soil microbiota. This requires using plant growth-promoting microorganisms (PGPMs) contributing in a range of biological events in the soil system to render it dynamic and robust for crop growth. The potency of PGPMs, their viable alliance with the host plant, and stable dynamics in the rhizosphere are explicitly linked to constantly fluctuating climatic conditions and other host-associated properties, which can highly influence the microbial growth, stability, and activity during the interaction of microbes with plant and other microbial community members. The three mechanisms stated earlier in this section are frequently interconnected and actively collaborate with the implementation of inoculants to shape soil features, even though their outcomes may vary. Since climatic variation is a worldwide issue, regulation and registration protocols for sustainable technologies that could help mitigate the problem should be simplified. Moreover, countries should be versatile or at the very least adopt simpler laws for registration and transportation of national and foreign microbiological techniques.

In addition to generating a more cost-effective and environmentally sustainable strategy to boost the supply and access of nutrients and protection from soil-borne diseases, PGPRs are the major protagonists in efforts to retain soil health (Lyu et al., 2020). Green agricultural practices limit the administration of chemical pesticides and mineral fertilizers. Therefore, in the view of sustenance of crop vigor and productivity eventually safeguarding from abiotic stressors, agriculture is increasingly reliant on biological pest management and organic fertilizers. In this perspective, we first presented a comprehensive outline of the current global status of salt stress together with its adverse repercussions on the environment and economy. Secondly, we discussed the potential advantages and signaling mechanisms of PGPR inoculants for climate change alleviation and adaptation, as well as to what extent these have been evaluated in fields. Then, we uncovered various advanced defense mechanisms to elucidate plant–microbe interactions at the molecular level and explore the potential molecular alteration by integrating them into a framework of high-throughput and omics technologies. Finally, we discussed the challenges and need for future research grasping the legacy of microbial inoculation in the context of climate change.

## 2 Data collection and analysis

**Figure 1:**
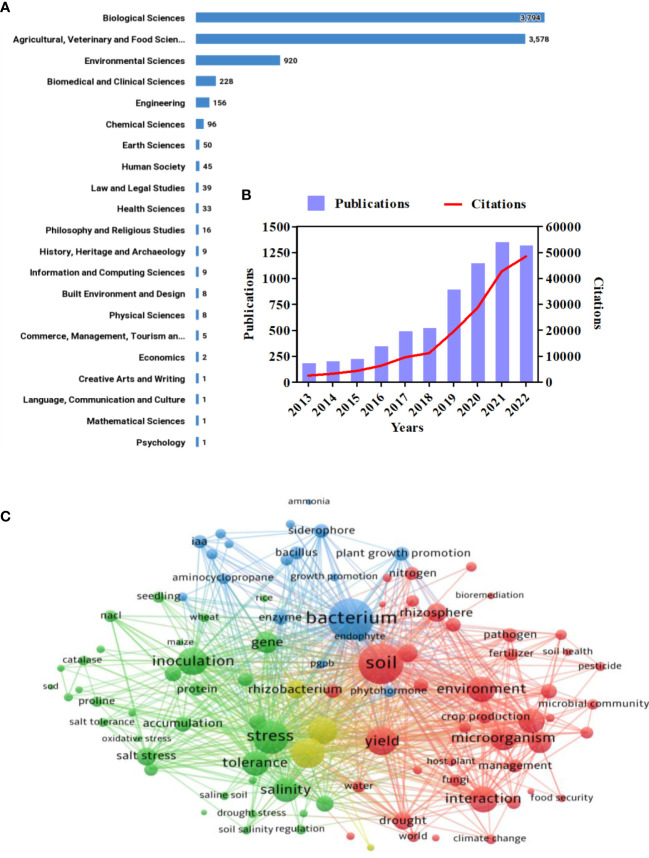
Scientometric analysis of the role of PGPRs in the amelioration of salinity stress. **(A)** Number of publications in different research categories. **(B)** Number of publications and citations in the literature on the application of PGPR in salt stressed agriculture from 2013 to 2022. **(C)** Overlay visualization of bibliometric network map of the most cited keyword associated with PGPR under salt stress representing four clusters: cluster 1 (green), cluster 2 (blue), cluster 3 (red), and cluster 4 (yellow).

The data consolidated in this investigation were extracted from the Web of Science Core Collection, providing a baseline for subsequent software processing and visualization. The data were exported on 10 November 2022, using the search terms “salt stress or salinity,” “plant,” “agriculture,” “microorganisms,” and “PGPR” in the dataset. Approximately 7,375 results were retrieved, which were then further filtered by year (from 2013 to 2022), yielding almost 6,680 results. Depending on the Australian and New Zealand Standard Research Classification (ANZSRC) 2020, **Figure 1A** illustrates the categorization of all published articles into different research fields. The year-wise distribution of articles published between 2013 and 2022 related to PGPR under salt stress, along with their citations, is shown in **Figure 1B**. A sharp increase in both publications and citations was observed in 2019, with the highest publication year being 2021 and the highest citation year being 2022. For scientometric analysis in this review, we utilized VOSviewer 1.6.18. A co-occurrence bibliometric map was constructed based on a keyword search employing the co-citation analysis theory and pathfinder network scaling (**Figure 1C**). Four clusters were identified in the network map: cluster 1 (green), cluster 2 (blue), cluster 3 (yellow), and cluster 4 (red). It is apparent that there have been more investigations between clusters 1 and 4 than between clusters 2 and 3. The keywords “bacterium,” “soil,” “stress,” “tolerance,” “inoculation,” “yield,” and “interaction” were the most frequently searched in the database. This implies that the most recent research has focused on bacterial inoculation to promote crop yield and tolerance of salt-stressed plants.

## 3 Salinity status in India and the world

Soil salinity has become a prime issue globally because of the damage it does to crop yield and its continuity. It has been known for ages that soil salinity exists and yet mankind and salinity have coexisted for many years. The life of people all over the world is negatively impacted by an excessive concentration of soluble salts in soils, which also have an unfavorable effect on agricultural fields and crops (Shahid et al., 2018). Identification of such soils can be done when certain alterations can be observed over its surface, like salty spots (bare land with white salt crust with the appearance of salt crystals). Other reasons that fuel the cause are prolonged water logging after rainfall, the deterioration and collapse of roads, and the degradation of ground and surface water, which negatively affects cattle and people (Srivastava et al., 2019).

Understanding the variations between salty and less saline soils is absolutely essential; exchangeable sodium percentage (ESP)<15.0, pH 6.5–8.5, electrical conductivity (EC)<4.0 dS m^−1^, and an equivalent distribution of anions and cations are all characteristics of a normal soil (Sharma, 2014). A saline soil, however, has an ESP of about 15.0, sodium adsorption ratio (SAR)<13.0 (at 25°C), EC >4.0 dS m^−1^, and pH<8.5 (Allotey et al., 2008; Etesami, 2018).

There are more than 100 nations where soil salinity is dynamically increasing, and no continent is entirely free of it. In the world, salt has an impact on about 831 million hectares (Mha) of the land surface, out of which 434 Mha (52.2%) of this region is sodic, while 397 Mha (47.8%) is saline. Asia, the Pacific, and Australia collectively have the biggest zone (30%) that is impacted by salt on a regional level (Sharma, 2014). About 45 Mha of irrigated land (20%), which produces three quarters of the food worldwide, is also doomed due to salinity (Shrivastava and Kumar, 2015). Of the 1.5 billion hectares of fertile land in the globe, 77 Mha (5%) have high salt content and do not support excellent yields, while salinity has choked 20% of the irrigated agricultural land (Selvakumar et al., 2014). The United Nations’ Food and Agriculture Organization claims that the land (about 1%–2%) that may be utilized for agriculture is lost to soil salinization every year; this issue is exacerbated in semiarid and arid regions (FAO, 2002). Approximately 50% of the 250 Mha of irrigated land worldwide, according to the FAO, 1988, has already experienced salinity stress and soil saturation issues and 10 Mha of irrigated land are abandoned each year as a result of salinization.

Regarding the state of salt-influenced soils on the world map, one of the primary factors contributing to desertification in the European Union is soil salinization, which impacts an estimated 1 Mha, primarily in the Mediterranean nations. The highest area of salt-affected soils on a continental scale was found in Asia (including the Middle East) with 7.14 M km^2^, followed by Africa with 2.292 M km^2^ and then by Australia and Oceania with 1.313 M km^2^, South America with 0.527 M km^2^, North America with 0.422 M km^2^, and lastly Europe with 0.024 M km^2.^ China, Australia, Kazakhstan, and Iran were the top-ranking nations with regard to the salt-affected areas, with 211.74 Mha, 131.40 Mha, 93.31 Mha, and 88.33 Mha, respectively (Hassani et al., 2020). There is a serious impact on irrigated land in Spain: about 3% of the 3.5 million hectares are in grave danger as salinity significantly lowers their capacity for agriculture, and another 15% is also facing the same fate (Stolte et al., 2015). As a consequence of the anticipated rise in cultivable lands and growing shortage of high-quality water in the Mediterranean region, field deterioration related with soil alkalinity may develop at accelerating rates in the future decades (Bowyer et al., 2009). As a global status in Mha, about 14% of the areas near East, 15% of North America and Africa, 16% of Latin America and Europe, along with a collective 24% of Asia, Pacific, and Australia are the regions with a preponderance of soil salinity (Srivastava et al., 2019).

For the lands in India, over 9.38 Mha of soil have salt effects through salinity, sodicity, or both; of this, 5.5 Mha are naturally salinized (Jaleel et al., 2008). There are more salt-affected soils in this country’s major states than ever before. The states with the most salt-affected land include Gujarat (2.23 Mha), Uttar Pradesh (1.37 Mha), Maharashtra (0.61 Mha), West Bengal (0.44 Mha), and Rajasthan (0.38 Mha). A ballpark figure of India’s salt-affected areas, which is about 80%, is positioned in these states. The most overwrought state is Gujarat, with 1.68 Mha of affected region, which is followed by West Bengal with 0.44 Mha of land and by Rajasthan (0.19 Mha) and Maharashtra (0.18 Mha). Scenarios of Uttar Pradesh dwell the impinging of salinity with 1.37 Mha of its land, out of which 0.02 Mha bears the troubles of salinity and the other 1.35 Mha with sodicity. The Agricultural land of Uttar Pradesh with the boon of crop productivity mainly faces the repercussion of salinity. On the other hand, the scenario is different for states like Kerala, Orissa, and West Bengal, along with Andaman and Nicobar Islands, as these predominantly face the severity of salinity (Singh, 2018). The condition of India’s groundwater resources (25%) is that they are saline and brackish, which hits the states of Haryana (holding 62% resources) and Rajasthan (holding 84% of resources) with tyrannical effects of salinity (Sharma, 2014). As a result, the quality of irrigation water in these states is often poor, and the consistent application of this water for irrigation contributes to the salinity and sodicity issues. The major factor contributing to the establishment of salt stress in the canal commands of India is attributed to having an inadequate drainage system by 2025; India is expected to procure 11.7 Mha of sodic and salinity-affected land (Sharma and Chaudhari, 2012).

The exorbitant deposition of water-soluble salts generates a condition known as soil salinity. It typically contains NaCl from table salt. Salts include numerous compounds of sodium, potassium, calcium, magnesium, sulfate, chloride, carbohydrate, and bicarbonate. Based on the salt concentration, salt-affected lands are frequently categorized as saline, sodic, and saline–sodic. The biggest repercussions of salinization on plant development are their damaged water uptake system. Despite the availability of moisture in soil, plants struggle and die since they are unable to imbibe sufficient water. As published in the 2018 Inter-governmental Science Policy Platform on Biodiversity and Ecosystem Services (IPBES) report, it is accurate on a worldwide level that an average of 190 million acres are entirely wasted, 150 million acres are degraded, and 2.5 billion acres are negatively impacted by soil salinization.

## 4 Effect and causes of soil salinity

### 4.1 Adverse implications of soil salinity on plant

Retention of soluble salt in soil naturally occurs or is due to improper human conduct, especially agricultural practices. Furthermore, some soils are originally salt affected due to inadequate salt elimination and dissolution. Causes of soil salinization include high evaporation rate, resulting in the addition of salts to the ground surface; dry and low rainfall regions when excessive salts are not washed from the land; insufficient drainage or water logging when salts are not washed owing to the absence of water transportation; ablation of deeply rooted plants that elevates the water table; irrigation with salt-rich water, which increments the soil salt content; geological deposit leaking and groundwater intrusion; sea level rise brought on by the seepage of sea salt into lower lands; seawater submersion followed by salt evaporation; coastal winds that transport salty air masses to neighboring locations; and improper fertilizer application when excessive nitrification speeds up soil salinization (Okur and Orçen, 2020).

The growth and production of crops are threatened by soil salinity, which also inhibits the sustainable growth of contemporary agriculture. More than one-third of the irrigated cropland in the world is impacted by soil salinity. Rising salinity levels in groundwater and inadequate drainage and irrigation systems are the main contributors to soil salinity. All of the major staple crops, including rice, wheat, and corn, accounting for the majority of human caloric intake, are glycophytes, which cannot finish their life cycles in soil with a NaCl content of more than 200 mM. Consequently, it is essential to enhance the salinity stress resistance level of crops to guarantee global food security. Comprehending how high salt concentration influences the morphological, biochemical, metabolic, physiological, and gene expression characteristics of crops is important to accomplish this aim. High salinity causes the apoplast to become alkaline, which prevents cell development. Rapid alkalinization factor (RALF) peptides that can alkalinize the apoplast *via* controlling H^+^ ATPases at the plasma membrane have been discovered (Zhao et al., 2020). The ability of high salinity to trigger the formation of mature RALF peptides suggests that RALFs are likely the mechanism through which salt stress causes the apoplast to become alkaline. The reduced photosynthetic efficiency under high salinity is primarily to blame for the inhibition of growth. K^+^ is a crucial nutrient for plants since it controls pH, chloroplast volume, and electron transport. Ionic, osmotic, and oxidative stressors are brought on by excessive Na^+^ and Cl^−^ buildup because it reduces K^+^ input into chloroplasts. Retrograde signaling pathways can transmit the chloroplast stress-related signals to the nucleus. One of the regressive signals is the generation of 1O_2_ brought on by high salinity, which leads to the photo-oxidative degradation of PSII. Executer (EX1), a nuclear encoded protein located in the thylakoid membrane of chloroplasts, is capable of sensing 1O_2_. EX1 is degraded more quickly as a result of stress-induced 1O_2_ release, and this degradation is reliant on an oxidative posttranslational alteration at the Trp643 residue in the EX1 DUF3506 domain. The most important and the initial stage in controlling gene expression is transcription. Early transcriptomic investigations in *Arabidopsis* suggested that hundreds to thousands of genes were altered by salt stress, based on the degree or duration of the treatment. Stomatal conductance (Gs), which causes considerable decrease owing to the salt-induced poor soil water potential, is a physiological reaction of the plant to the decreased water availability (or “physiological drought”) caused by salinity. Quinoa’s stomatal density is significantly reduced by salinity (by roughly 30%), and many other halophytic plants have shown a negative relationship among stomatal density and salt tolerance.

Salinity produces a multitude of physiological and biochemical problems in crops, such as lower soil water potential, particularly ion effects, ionic disequilibrium, and a greater deposition of reactive oxygen species (ROS) and OH^−^ ions. These ROS can damage plant tissue, alter DNA, disrupt cell membranes, and degrade lipids, proteins, and other bio-molecules. Because there is more Na^+^ in the soil solution, salt-affected lands evince greater Na^+^/K^+^ and Na^+^/Ca^2+^ ratios. Consequently, a decrease in K^+^ and Ca^+2^ absorption results in the suppression of cellular function, instability of cell membranes, and interference with enzymatic activity. Rice (*Oryza sativa* L.) and wheat saw increased growth as a result of selenium administration through enhancing stress tolerance systems including antioxidant and secondary metabolite pathway; selenium exhibited control of crop growth (Kamran et al., 2019). The physiological characteristics of cereal crops including wheat (*Triticum aestivum* L.) and mung bean (*Vigna radiata* L.) are reduced due to soil salinity stress (Khan et al., 2014; Ahanger and Agarwal, 2017; Elkelish et al., 2019). Ion imbalance in the soil and plant is brought on by the entry of Na^+^ and Cl^−^ ions, and this imbalance in the plant’s ions may have serious physiological effects (Shahid et al., 2020).

An excessive salinity in the soil profile may result in physiological dryness as a result of decreased water absorption, salt buildup in the rhizosphere, a reduction in plant osmotic potential, and subsequent disruption of cell metabolic processes owing to ion toxicity (Safdar et al., 2019; Shahid et al., 2020). The normal plant metabolism may eventually be disrupted by these ROS because of changes to the structures of lipids, proteins, and nucleic acids (Garcia-Caparros et al., 2021). According to some reports, salinity-induced oxidative damage in the soil brought on by the accumulation of increased amounts of H_2_O_2_ may cause DNA breakage, chromatin condensation, apoptosis, and cell shrinkage (Kamran et al., 2019). Additionally, soil salinization results in acute oxidative damage to plant tissues, leading to the development of a sophisticated natural antioxidant defense in plants. The cell structural harm brought on by salinity-induced ROS is prevented by antioxidant enzymes. Se, like other heavy metals/metalloids, serves as a pro-oxidant at high concentrations, increasing the generation of ROS while also causing protein oxidation, lipid peroxidation, and genotoxicity (Debnath et al., 2021). Analogous to this, another study found that Se treatment as sodium selenite improves eggplant growth and yield at various soil salinity levels (Kamran et al., 2019). The Se (Na_2_SeO_4_) availability, however, decreased Na^+^ and enhanced root development, which may have increased the water supply to shoots and supported plant growth (Subramanyam et al., 2019). Higher Na^+^ ion concentrations in plant roots under salt stress decrease hydraulic conductivity, which eventually lowers relative water content (RWC). Salt-affected soils hinder the uptake, accumulation, and metabolism of nitrogen, which disrupts the biosynthetic process for proline, a molecular chaperone in charge of preserving protein integrity (Shafi et al., 2019). Hydraulic conductivity, water penetration, and porosity decrease when salt concentration in soil rises due to structural degradation (Minhas et al., 2021). High salt content has a number of harmful consequences on soil, including high pH, loss of organic matter, impaired nutrient cycling (C and N mineralization), nutrient scarcity, and toxicity of few predominant anions/cations (Sahab et al., 2021). The number of bacteria and actinobacteria colony-forming units (CFU) has significantly decreased. Additionally, *Azospirillum brasilense* adhesion to the roots of maize and wheat was diminished by high salinity. Excessive salinity altered the *nif* gene of *A. brasilense* (ATCC 29710), which is associated with roots and in charge of N_2_ fixation and nitrogenase activity (Sahab et al., 2021). Saline-irrigated agricultural soils have much lower concentrations of microbial activity. Additionally, they came to the conclusion that salinity has an influence on soil enzymes as well as microbial biomass carbon (MBC).

### 4.2 Soil salinity impact on economic status

The main economic price of salt stress is paid by farmers in the form of poor yields. In addition, economic expenses vary considerably country-wise and are greatly impacted by the cost of farmers’ input vs. profit they generate during a season with average rainfall. In broad-acre dryland agriculture, crop input costs might reach $300 per hectare. Under water-limited conditions, a crop yield of 3 tons per hectare will generate a gross revenue of $600 per hectare and a net return of 300 tons ha^−1^ at a good market price of, say, $200 per ton. Under saline conditions, when the crop production is dropped to 2 tons ha^−1^, the gross income falls to $400 ha^−1^ and the net return drops to merely $100 ha^−1^. Continuous loss from salt stress brought on by climate change or a rising water table implies that farming is challenging and that land use will be limited to pasture production of only salt-tolerant plants like halophytes. As a result, farmers receive somewhat reduced returns, yet cropping may still be profitable given the decreased input costs. Farmers live on the edge of profit or loss as a number of elements require additional expenditures. Hence, even a minute reduction in yield or any involuntary alteration in land usage may have disastrous effects (Munns and Gilliham, 2015).

## 5 Salinity stress, PGPR, and plant productivity: A Triangular conjecture

Nowadays, burgeoning urbanization is attributed to immense climatic shift, which is directly taking toll on crop production and its yield. Soybean is among one of the malleable crops with deleterious effects due to the actions of biotic and abiotic stresses prevailing in the environment. Innumerable solutions have been proposed to combat the adverse consequences of flourishing abiotic stresses including high temperature, salinity, and drought conditions; however, none of them garnered enough attention for implementation as PGPR. Microorganisms have been proven to be a viable solution in alleviating numerous abiotic stresses. Induced systemic tolerance (IST) refers to PGPR affected by physical and chemical alterations leading to increased resilience to abiotic stressors. The use of stress-resistant competitive consortia involving PGPB and rhizobia can undergo their mode of action to increase the sustainable crop productivity. PGPRs are reported to function as plant growth-promoting agents through two mechanisms (Numan et al., 2018). First and foremost, PGPRs trigger the metabolic pathways of the plant, such as catalyzing the synthesis of growth hormones, stimulating the antioxidant and siderophore production, and amplifying the nutritional storage of plants. Furthermore, PGPR themselves are responsible for the production of numerous phyto-hormones, for instance, auxins and cytokinins, facilitating development of roots along with shoots or refining nutrient supplement by phyto-pathogenic antagonism, N_2_ fixation, and mineral solubilization.

Enhancing salinity as a soil condition is the predominant factor in curbing the growth and yield of soybean crop. The wide spectrum of salinity-tolerant PGPR, comprising pivotal soil microbiota, interacts with the host legumes and aids in mitigating the detrimental repercussions of salinization as well as promotes soil health and efficiency. The halo-tolerant bacteria impart several PGP traits and benefit the crop with stress endurance during extreme salty soil conditions (Prittesh et al., 2020; Gupta et al., 2021; Kumawat et al., 2021). In a recent investigation, Peng et al. (2021) selected a consortium of three bacterial strains (*Pseudomonas* sp. P8, *Peribacillus* sp. P10, and *Streptomyces* sp. X52) inoculated in the roots of maize plants in salt-affected soil and witnessed accelerated plant growth due to the salt resisting ability of the bacterial consortium. Under salt-stressed conditions, majority of the rhizospheric bacteria secrete osmo-protectants (glutamate, trehalose, proline, proline betaine, and ectoine) to maintain their cytoplasmic osmolarity (Kumar and Verma, 2018). The periplasmic space of bacteria has a large amount of highly anionic polysaccharides referred to as membrane-derived oligosaccharides, which are too large to diffuse through the porin proteins and presumably operate to preserve the turgor pressure of their periplasm (Sandhya et al., 2017). Numerous studies have been undertaken over the past 20 years demonstrating the beneficial role of rhizobacteria on plant growth *via* eliciting salinity stress coping mechanisms in varied crops (paddy, rice, chickpea, wheat, groundnut, alfalfa, and tomato) (Banerjee et al., 2017; Zhang et al., 2018; Desoky et al., 2020; Nagpal et al., 2020; Kumawat et al., 2021).

## 6 Signaling mechanisms of salt stress in plants

**Figure 2:**
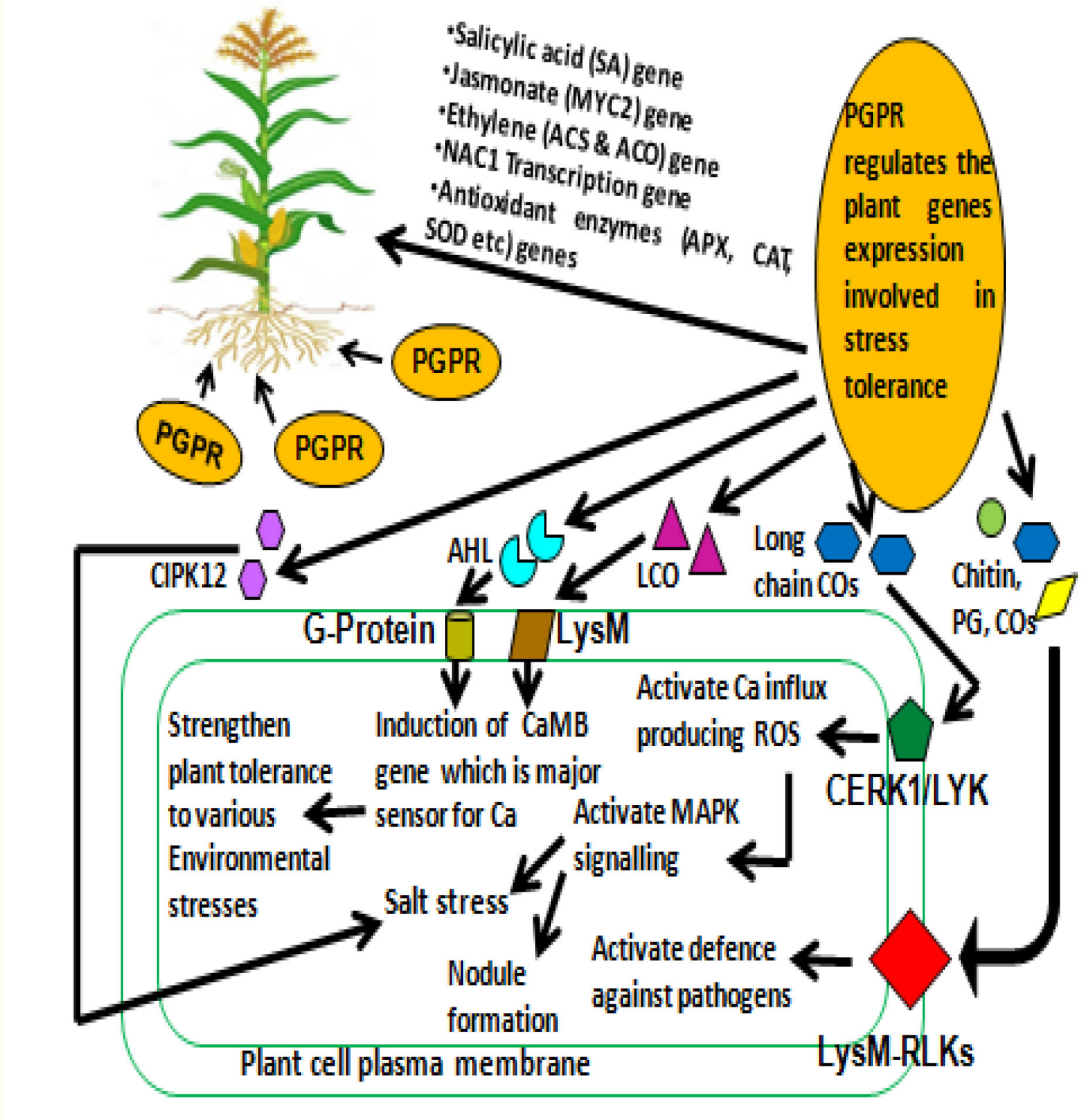
PGPR induced signaling pathway in plant to improve the stress tolerance PG, peptidoglycan; Cos, chitin oligomers; CERK1, chitin elicitor receptor kinase1; LYK, LysM receptor-like kinases; LysM-RLKs, lysin motif receptor-like kinases; LCO, lipo-chitooligosaccharide; LysM, lysin motif receptor; ROS, reactive oxygen species; MAPK, mitogen-activated protein kinase; CaMB, calmodulin-binding protein; Ca, calcium; AHL, *N*-Acyl homoserine lactones; CIPK12, CBL (calcineurin B-like) interacting protein kinase12].

Several varied physiological as well as biochemical changes are observed in plants that are induced to mitigate stresses. Salinity stress is one such example, which consequently invokes myriad modulations in genes relating to stress and their subsequent signaling pathways. Generally, plants inherited genetic traits to overcome salt stress or plants have developed a mechanism for salt expulsion that delays stress of salinity. Besides this, rhizospheric microbes, especially PGPR, contribute crucially in overcoming salt stress (Bhat et al., 2020). The understanding of these mechanisms associated with hyper-salinity stress signaling becomes all the more important when it occurs between plant and rhizospheric microbiome (Numan et al., 2018; Kumawat et al., 2022a). Generally, surface receptors first perceive the signal and, in turn, generate secondary messengers (inositol phosphates and ROS) that start the cascade of plant signal transduction. Gene expressions during stress are controlled by calcium ion modulation brought about by the secondary messengers that act upon calcium-dependent protein kinase (CDPK), microtubule-associated mitogen-activated protein kinase (MAPK), and protein phosphatases (Kumar et al., 2020). This involves a large amount of transcription factors of families such as bZIP, MYB, AP2/ERF, and NAC that modulate genes related to salinity stress signaling. These modulations are mostly exclusively posttranscriptional. A number of PGPB and endophytes have demonstrated induction gene of signaling under different types of stress (**Figure 2**).

MAPK and CDPK are two important pathways utilized by the plants to counteract environmental stress. MAPK is highly conserved (MAPK, MAPKK, and MAPKKK), and the mechanism of phosphorelay is utilized for signal transmitting. MAPK is activated by various signal molecules, and within these pathways, cross-talk may occur for better survival under stress. Generally, under stress conditions, cells respond to external environment quickly by the existence of proteins sensing calcium (CDPK and calmodulins) and by modulating cytoplasmic calcium ions. Under abiotic stress, CDPK modulates the production of ROS and abscisic acid (ABA) in plants. PGPRs have the ability to produce various metabolites/hormones [cytokinins, gibberellic acid, indole-3-acetic acid (IAA), zeatin, ABA, ethylene, etc.], which act as signal molecules to interact with plant roots and assist plant in overcoming stress (Jalmi and Sinha, 2022). Besides this, volatile organic compounds (VOCs) secreted by PGPR help in communication between the bacteria and plant root in the rhizospheric region (Ayaz et al., 2021; Jalmi and Sinha, 2022). Parallel to this, plants secreted root exudates in the rhizosphere, which also contain different types of signal molecules (like volatile and non-volatile compounds, and high- and low-molecular-weight compounds) that regulate microbial interaction and selectively inhibit/proliferate a particular microbial species. PGPRs contribute in providing resilience to salinity stress in plants by interfering with different mechanisms by regulating ion homeostasis and uptake. Thus, various genes/proteins (MAPK, RAB18, DREB, and CIPK12) of PGPR are involved in modulating the defense and biosynthesis of hormones for better adaptation of plant under salinity stress (Ilangumaran and Smith, 2017; Jalmi and Sinha, 2022).

A mechanism involving enhancement of stress tolerance in wheat is demonstrated by using *Arthrobacter protophormiae* and *Dietzia natronolimnaea*, two prominent PGPRs. This was done by intervention in one of the regulating elements of ethylene signaling pathway and DREB2—the CTR1 (constitutive triple response). It is *via* the modulation of this CTR1 that enhanced salinity tolerance was observed. Another study involving *D. natronolimnaea* found that it was responsible for induction of expression of TaMYB and TaWRKY genes in wheat (Bharti et al., 2016). *Enterobacter* sp. has also been known to increase the expression of salinity stress response genes in *Arabidopsis* under high salinity. These genes include RD29A, RD29B, DREB2B, and RAB18 (Kim et al., 2014; Barnawal et al., 2017; Joshi and Rajkumar, 2019).

Recently, Ali et al. (2022) presented that application of salt-resistant *Enterobacter cloacae* in maize upregulated salinity stress-related genes (SOD and APX). Salinity stress signals may sometimes be easily confused for other responses mainly due to their similarities with drought stress signals and the dubious efficacy of stress-induced genes due to the influence of epigenetic processes like DNA methylation and posttranslational modifications (Dietz et al., 2010; Golldack et al., 2013). The expression, however, also varies as the type or variety of plant changes. Molecular biology and advancement in genomics may hold a promising future for understanding these responses with perfect clarity.

## 7 Microbial resistance and resilience under salinity stress

External environmental stressors may inevitably lead to variation in the microbial community dynamics. The ability of that community to avoid such changes is referred to as resistance, whereas resilience is the capacity of a community to regain its initial state once the stress is released and favorable conditions resurface (Allison and Martiny, 2008; Kumar et al., 2020). It is the response and modifications of rhizospheric diversity that make it imperative to understand these changes. Microbial attributes, community structure, and functions are all affected by salinity stresses; however, if a community exhibits a high level of functional diversity, it may mean that multiple processes may run simultaneously at the same rate. A new bacterium, capable of surviving the stress imposed, may replace a pre-existing taxon and continue the same metabolic functions. In this way, the health of soil higher in salt content can be upscaled by growth of salinity-resistant communities. Additionally, these communities also help maintain various ecological functions along with sustaining and eventually promoting plant growth (Kumar and Verma, 2018; Kumar et al., 2020; Kumawat et al., 2022b).

**Figure 3:**
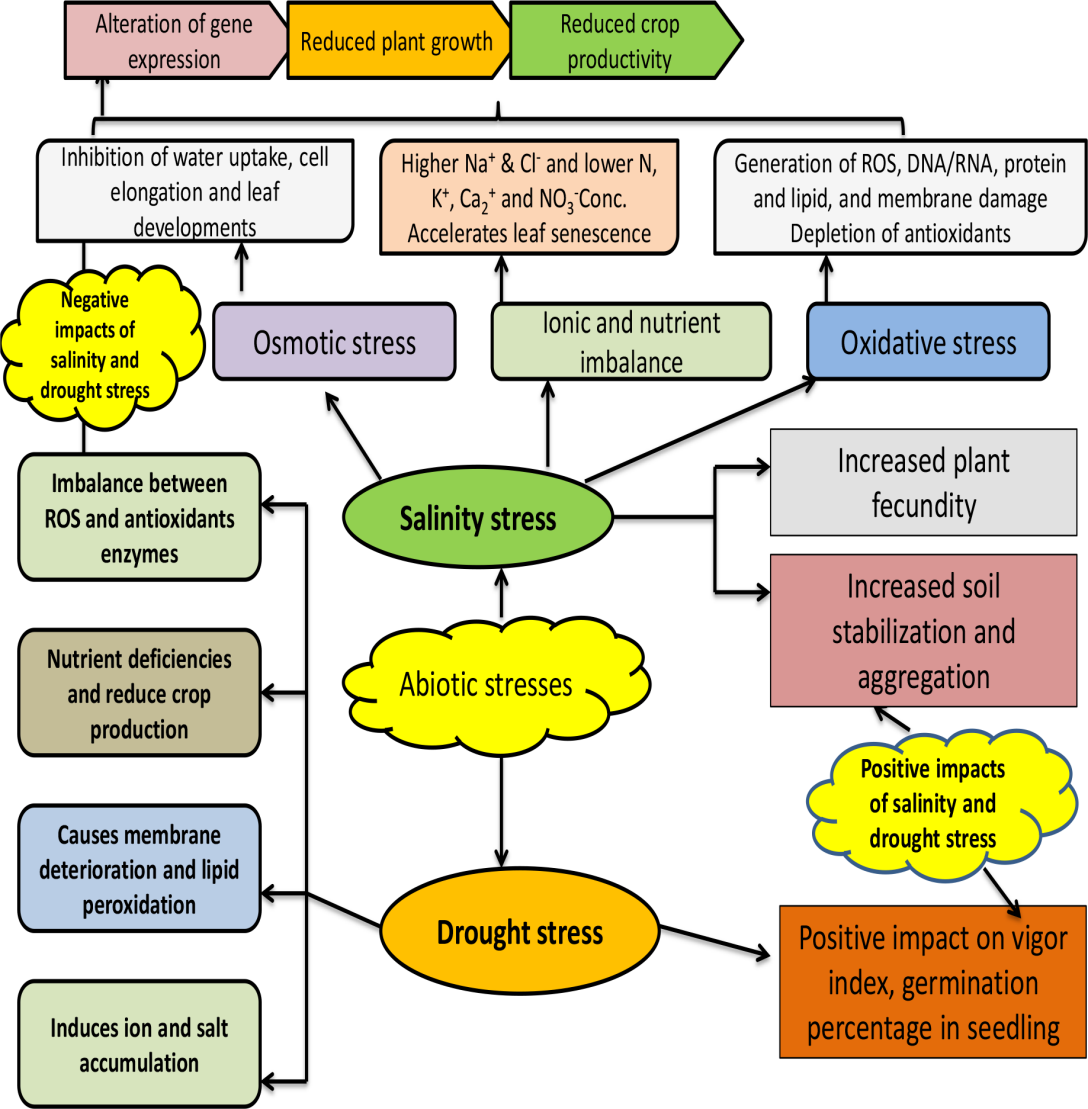
Beneficial and harmful impact of abiotic stresses (salinity and drought) on plant growth promotion under intensive cropping systems.

These halo-tolerant bacteria adopted various strategies to overcome salinity stress (**Figure 3**). These bacteria have a sodium ion export system that expels the excess sodium ions entering the microbial cell as high concentrations of sodium ion damage the microbial cell (Xu et al., 2022). Beside sodium ions, some halo-tolerant cells accumulate potassium/chloride ions to overcome salt stress (Kraegeloh et al., 2005; Zhang et al., 2018). In another strategy adopted by some halo-tolerant cells, the cell wall is stabilized by replacing glucose in the cell wall with lacto-proteins that impart negative charges, which helps in overcoming high pressure (Hamlet and Muller, 2013). Some salt-tolerant strains produce anionic phospholipase, which protect the microbial cell from high osmotic pressure and also help in maintaining water in the plasma membrane. Furthermore, compatible solutes (amino acids and sugars) are produced by many halo-tolerant cells, which helps in maintaining the concentration of electrolytes and water content in the cells under high osmotic pressure (Krulwich et al., 2011; Shivanand and Mugeraya, 2011). Thus, the presence of salt-tolerant microorganisms with PGPR attributes in the rhizosphere is very helpful in mitigating salt stress in plant growth.

Mahmud-Ur-Rahman Naser et al., 2022 isolated *Brevibacterium sediminis* from rice (*O. sativa*), which tolerate 12% NaCl stress. These bacteria showed enhanced growth of different rice seedlings tested. Ali et al. (2022) studied the salt tolerance of procured bacterial cultures and found that isolate *E. cloacae* PM23 was able to tolerate 3M sodium chloride concentration and this isolate also showed exopolysaccharide, ACC deaminase, siderophore, and IAA production. The application of *E. cloacae* PM23 in the tested maize plants showed increased content of flavonoid, total phenolics, proteins, soluble sugars, water content, and scavenging capacity of radicals. Halo-tolerant bacteria like *Staphylococcus epidermidis* not only have been known to survive in high salt concentrations (up to 30%) but also have plant growth-promoting properties. These bacteria use a number of mechanisms like production of extracellular proteases and activation of Na^+^ and H^+^ antiporters, along with accumulation of compatible solutes for osmo-regulation to overcome the effect of increased salinity. Instances where genes from halo-tolerant bacteria were used for development of transgenic plants have been common and usually a successful approach towards imparting salinity resistance in plants. One such example is the improved salinity tolerance in tomato (*Lycopersicon esculentum*) where synthesis of glycine betaine was induced using the codA gene derived from *Arthrobacter globiformis* that encodes for choline oxidase. Bharti et al. (2016) demonstrated that SOS1 and SOS2, which are two salt overly sensitive (SOS) pathway-related genes and part of the necessary transcriptional machinery for salt tolerance, were modulated by inoculation of *D. natronolimnaea*. The exercise also resulted in enhanced gene expression of various antioxidant enzymes like ascorbate peroxidase (APX), catalase (CAT), and superoxide dismutase (SOD). This was observed in addition to higher PGPR content in wheat plants. Despite still being in its infancy and in dire need of further research, the application of these stress-tolerant microbes has been proven to not only enhance plant and soil health but also improve the microbial community.

## 8 Rhizospheric microbiome as a salinity-alleviating agent

**Figure 4:**
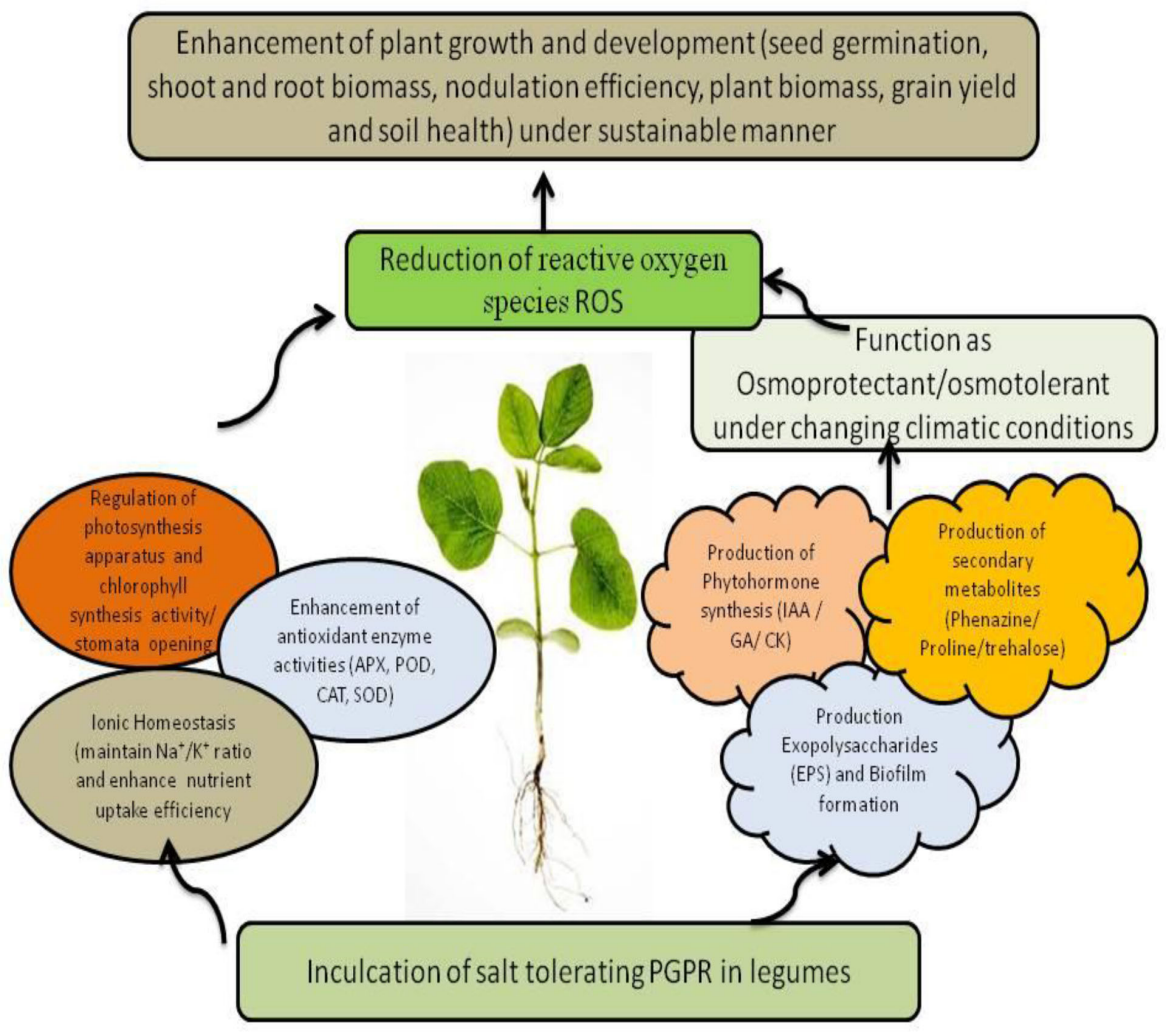
Possible events of PGPR-mediated salt tolerance mechanisms in plants under changing climatic conditions.

Rhizospheric microbiomes heavily colonize the plant roots due to the availability of a nutrient-enriched biosphere comprising amino acids, carbohydrates, fatty acids, and organic acids acting as a passageway for the attraction of multiple microorganisms that thrive in this rhizosphere by utilizing root exudates. This dynamic zone around roots facilitates the microbial synthesis of compatible solutes, enzymes such as 1-aminocyclopropane-1-carboxylate (ACC) deaminase, and cell wall-degrading enzymes ions, as well as other secondary metabolites including phyto-hormones such as auxins, cytokinins, gibberellins, and ethylene, which augment plant growth and defense system under saline stress conditions (**Figure 4**) (Tiwari et al., 2022). Microbes reduce stress in plants by controlling nutritional and hormonal equilibrium and inducing systemic tolerance to stress (**Table 1**) (Kumawat et al., 2022c).

**Table 1:** Mitigation of salt stress and plant growth enhancement through plant growth-promoting rhizobacteria (PGPR).

Plant species	PGPR	Response/effect of PGPR inculcation	References
Rice (*Oryza sativa*)	Bacillus aryabhattai	Improved salt tolerance ability *via* exo-polysaccharide production	Sultana et al. (2020)
	*Alcaligenes faecalis, Ochrobactrum* sp., and *Bacillus pumilus*	Increased photosynthetic activities as well as plant biomass and yield enhanced	Bal et al. (2013)
	*Bacillus* sp.	Enhanced essential nutrient solubilization, exopolysaccharide production, and improved emergence count as well as seedlings growth	Mishra et al. (2017)
	*Bacillus tequilensis* and *Bacillus aryabhattai*	Improved photosynthesis activities, transpiration rate, and crop productivity	Sultana et al. (2020)
	*Pseudomonas* strains	Increased plant height and leaf area index as well as improvement in crop productivity and soil health	Sen and Chandrasekhar (2014)
	*Enterobacter* sp.	Enhanced the biosynthesis of *SOD*, *CAT*, and *GSH*	Sagar et al. (2020)
	*Bacillus pumilus*	Decreased Na^+^ accumulation and improved plant growth	Khan et al. (2016)
Wheat (*Triticum aestivum L*.)	*Arthrobacter protophormiae* (SA3) and *Dietzia natronolimnaea* (STR1)	Increasing IAA, reducing ABA and ACC level, and adjusting expression of ethylene signaling regulatory compartment (CTR1) pathway and DREB2 transcription factor	Barnawal et al. (2017)
	*Pseudomonas azotoformans* FAP5	Biofilm development improved morphological and physiological attributes	Ansari et al. (2021)
	*Bacillus safensis* and *Ochrobactrum pseudogrignonens*	Enhanced antioxidant signaling and reduced chloroplast as well as membrane injury	Sarkar et al. (2018)
	Alternaria alternata	Improved photosynthesis *via* increasing antioxidant enzymes	Qiang et al. (2019)
	*Azospirillum* sp.	Enhanced photosynthetic pigment and plant growth	Zarea et al. (2012)
	*Pseudomonas fluorescens*	Promoted plant growth	Safari et al. (2016)
	*Pseudomonas fluorescens, Serratia liquefaciens, Bacillus subtilis*, and *Bacillus megaterium*	Increased Chl content and photosynthetic activities	Fathalla and Abd el-mageed (2020)
	*Serratia marcescens*	Increased shoot length, fresh weight and Chl content	Singh and Jha (2016)
Maize (*Zea mays*)	*Azotobacter* sp.	Maintained higher K^+^/Na^+^ ratios	Rojas-Tapias et al. (2012)
	*Bacillus megaterium*	Enhanced plant growth, roots, and root hydraulic conductivity	Marulanda et al. (2010)
	*Pseudomonas* sp.	Enhanced osmolytes synthesis	Fazal and Bano (2016)
	*Pseudomonas fluorescens, Pseudomonas putida*, and *Azotobacter vinelandii*	Increased plant height, leaf area, and Chl a	Abd El-Ghany et al. (2015)
	Bacillus HL3RS14	Increased weight of roots and shoots *via* production of IAA, proline, and glycine betaine	Mukhtar et al. (2020)
	Gluconacetobacter diazotrophicus	Increase plant biomass and chlorophyll content	Tufail et al. (2021)
	*Klebsiella variicola* F2, *Raoultella planticola* YL2, and *Pseudomonas fluorescens* YX2	Induced accumulation of glycine betaine and choline led to decline in water loss	Gou et al. (2015)
Chickpea (*Cicer arietinum*)	*Azospirillum* sp.	Improved phosphate solubilization, plant growth due to reduced salinity stress	Patel et al. (2012); Nagpal et al. (2019)
	*Pseudomonas putida*	Reduced salinity stress, improved plant growth promotion	
Soybean (*Glycine max*)	*Glomus etunicatum*	Enhanced P, K, Zn and proline content in roots. Increased root development and mineral concentration	Sharif et al. (2007)
	*Pseudomonas fluorescens, Pseudomonas putida*, and *Bacillus subtilis*	Enhanced osmolytes and POD synthesis	Abulfaraj and Jalal (2021)
	*Paenibacillus mucilaginosus*	Volatile organic compounds produced by bacteria reduced Na^+^ ions in root and shoot and increased proline content in root	Ma et al. (2018)
	Pseudomonas pseudoalcaligenes	Improved plant health parameters *via* production antioxidant enzyme, proline contents in shoots and roots	Yasmin et al. (2020)
	Aspergillus flavus	Increased antioxidant enzyme activity and chlorophyll content	Asaf et al. (2018)
	*AMF* and *Bradyrhizobium* *japonicum*	Increased yield and protects from stress *via* upregulation of CAT and POD activity	Sheteiwy et al. (2021)
	*AMF* and *Rhizobium* sp.	Improved plant health and microbial diversity in soil triggered CAT, proline, and IAA production	Igiehon et al. (2021)
Pea (*Pisum sativum*)	*Acinetobacter bereziniae, Enterobacter ludwigii*, and *Alcaligenes faecalis*	Enhanced osmolytes soluble sugar and proline synthesis	Sapre et al. (2021)
	*Brachybacterium saurashtrense* and *Brachybacterium casei*	Had higher K^+^/Na^+^ ratio and higher Ca^2+^, phosphorus, and nitrogen content	Shukla et al. (2012)
	*Bacillus sp.*	Improved plant growth and photosynthesis *via* antioxidant enzyme and AAC and siderophore production	Gupta et al. (2021)
	*Rhizobium leguminosarum* bv. *viciae*	ACC deaminase increased nodulation, shoot biomass, water use efficiency, and nutrient uptake	Belimov et al. (2019)
Tomato (*Solanum lycopersicum*)	*Bacillus megaterium*	Increased biosynthesis of phytohormones and polyamines Increased root, shoot, and leaf growth	Nascimento et al. (2020)
	*Enterobacter* sp.	Enhanced ROS scavenging activity	Kim et al. (2014)
	*Glomus mosseae*	Increased *CAT, SOD, APX*, and *POD* activity in roots. Reduced cell membrane damage and increased plant growth under salt stress conditions	He et al. (2007)
Alfalfa (*Medicago sativa*)	*Bacillus megaterium*	Increased nodule formation, and enhanced production of IAA and ACC deaminase	Chinnaswamy et al. (2018)
	*Hartmannibacter diazotrophicus* and *Pseudomonas* sp.	Increased plant height, leaf number, Chl a, Chl b, total Chl, carotenoids content, and photosynthesis rate	Ansari et al. (2019)
Sunflower (*Helianthus* *annuus*)	*Pseudomonas fluorescens*	Stimulated plant growth, enhanced IAA, pyocyanin, and phosphate solubilization	Tewari and Arora (2016)
	*Rhizophagus irregularis* and *Chryseobacterium humi*	Reduced nutrient imbalance and improved K^+^/Na^+^ ratios in plant tissues	Pereira et al. (2016)
Barley (*Hordeum vulgare*)	*Hartmannibacter diazotrophicus*	Increased water content in the root system. Improved shoot and root dry weight and plant growth	Suarez et al. (2015)
Oat (*Avena sativa*)	*Klebsiella* sp.	Enhanced relative water content, increased shoot and root dry weight and plant growth	Sapre et al. (2018)
Arabidopsis (*Arabidopsis thaliana*)	*Bacillus megaterium*	Improved root and shoot growth	Chinnaswamy et al. (2018)

### 8.1 Phyto-hormone production and ACC deaminase activity

Rhizo-microbiome synthesizes phyto-hormones in low concentration, which act as chemical messengers to regulate the metabolic routes of growth simulators in plants and alter the root morphology when exposed to stress. Several published research reports support the hypothesis that phyto-hormone production is among the major plant stress alleviation mechanisms adopted by microbes. Auxins and IAA stimulate cellular division, expansion, and differentiation in plant roots in addition to the development of lateral roots, both of which aid in the plants’ nutritional uptake with water retention (Ilangumaran and Smith, 2017). Haroon et al. (2021) published an article in which the root-associated halo-tolerant PGPRs *Bacillus megaterium, B. tequilensis*, and *Pseudomonas putida* have been utilized *via* seed priming to ameliorate stress-induced deterioration and upsurge growth of salt-stressed wheat plants, through IAA synthesis. They reported an overexpression of salt-sensitive (*SOS1* and *SOS4*) genes involved in salt stress mitigation.

Cytokinins are N^6^-substituted adenine analogs with over 40 structural variables presently defined, including zeatins (isoprenoid functionality) and topolins (aromatic functionality). They pertinently serve in the modulation of plant acclimatization to biotic and abiotic stress, as well as promote plant growth and development through cell division and elongation, shoot growth, nutrient uptake, senescence prevention, vascular development, gametophyte development, and other mechanisms (Fahad et al., 2015). Intensive research over the last decade has gradually revealed a key significance of CKs in the modulation of salt resilience in numerous model plants. High salt concentrations normally do not impair the proficiency of root-associated microorganisms to generate phyto-hormones. Multiple investigations have established the beneficial impacts of microbes linked to plants and CK generation improves plant growth in the saline ecosystem. For example, *Bacillus subtilis* enhanced the content of CKs in salt-stressed plants. The rhizospheric inoculation with CK-producing *B. subtilis* preceded an upregulation in the expression of a CASP-like protein gene 4D1, a part of the Casparian strip membrane domain protein group, resulting in an accelerated emergence of Casparian bands in root endodermis and improved growth of durum wheat variety in salt (Martynenko et al., 2022). Elevated protein tyrosine nitration (PTN) is a well-established plant mechanism to salinity. Salinity influenced tomato seed coating with the bacteria *Achromobacter xylosoxidans* in combination with irrigation with an extract of the marine algae *Enteromorpha intestinalis* suppressed PTN while augmenting cis-zeatin-type and iso-pentenyl adenine (iP) cytokinins. PTN action is inversely related to CK levels in plants under stress, specifically cis-zeatin-type-cis-zeatin (cZ) with cis-zeatin riboside (cZR) and iP, offering increased resistance toward salinity (Zizkova et al., 2015).

The ABA pathway is essential for plants to regulate salt stress. Osmotic stress activated by salt stress causes ABA accumulation in plants, which stimulates acclimation response. ABA affects many plant processes, including cell differentiation, seed germination, organ development, stomatal mobility, and production of stress-related proteins and metabolites. These mechanisms are critical in reducing plant damage induced by stress (Song et al., 2022). Plant growth-promoting rhizo-microbiome has been proven to be efficient in plant salt stress alleviation *via* ABA regulatory management. *B. pumilus* MAK9 and an endophytic bacterium, *Curtobacterium* sp. SAK1, increased salinity stress tolerance in Chinese cabbage and soybean, respectively, while increasing plant growth parameters (Khan et al., 2019a; Khan et al., 2022). A wide range of stress response genes are regulated by ABA. Different genes associated with the regulation of the ABA pathway under salt stress have indeed been discovered thus far. *Arthrobacter woluwensis* AK1 synthesized phyto-hormones such as IAA and ABA, which upregulated the expression of stress-resistance genes (*GmST1* and *GmLAX3*) in soybean plants grown in 300 mM NaCl (Khan et al., 2019b). Similarly, inoculation of *Stenotrophomonas* sp. SRS1, isolated from salt-tolerant *Carex distans* (distant sedge) roots, into *Arabidopsis thaliana* and *Solanum lycopersicum* (cherry tomato MicroTom) prompted the overexpression of the salinity-associated gene markers *RD29A* and *RD29B*, as well as the *ABI4::GUS* expression, although growth was terminated in the presence of salt, where there was no abolishment under unstressed conditions (Manh et al., 2022). ABA also influences the water dynamics by lowering transpiration *via* ABA-induced stomatal closure while increasing water circulation *via* ABA-induced improvements in activation of aquaporins. Salinity decreases water availability, manifesting in poorer bulk and relative water concentration in plants. *B. subtilis* IB22 treatment ramped up root ABA level while decreasing shoot ABA deposition by upregulating (*HvNCED2*) and downregulating (*HvCYP707A1*) ABA catabolic genes in barley cultivated at 100 mM salt concentration (Krishnamoorthy et al., 2022). In salt-stressed *Arabidopsis*, *Enterobacter* sp. promotes the expression of salinity stress-responsive genes (*RD29 A, RD29 B*, and *RAB18*), ABA-responsive element regulons, *DREB2B*, and dehydration-responsive elements, resulting in ABA-independent activation (Kim et al., 2014).

Ethylene is a gaseous hormone. Its biosynthesis involves a relay process that begins with the transformation of S-adenosyl-methionine (SAM) to 1-aminocyclopropane-1-carboxylic acid (ACC) utilizing ACC synthase and ends with the formation of ethylene from ACC by the action of ACC oxidase. In low concentrations, ethylene positively controls seed germination, root elongation, and flowering. However, when under stress, ethylene levels rise, which has a negative impact on plant growth (del Carmen Orozco-Mosqueda et al., 2020). Several rhizomicrobes synthesize an enzyme named 1-aminocyclopropane-1-carboxylic acid deaminase (ACCD) in their cytoplasm, which catalyzes the breakdown of ACC into ammonia and alpha keto-butyrate. Moreover, ACCD nullifies the IAA feedback inhibition triggered by elevated ethylene production in plants. ACCD-producing microbes can regulate ACC levels in the rhizosphere and phyllosphere by acquiring plant-released ACC or inside plant tissues (e.g., the endosphere and root nodules), thus further explicitly restricting ACC activities, consequently curtailing ethylene production, ameliorating stress, and stimulating plant growth in challenging circumstances. ACCD-producing halo-tolerant *Brevibacterium linens* RS16 promoted plant growth traits in rice and red pepper cultivated amid salt stress (Chatterjee et al., 2018). *E. cloacae* isolated from *Ziziphus nummularia* enhanced wheat plant development by raising ACCD activity under salinity stress (200 mM). Likewise, the endophytic *Paenibacillus xylanexedens* PDR6 from *Phoenix dactylifera* roots displayed ACCD activity and suppressed the ethylene level during salinity condition (Singh et al., 2022a; Singh et al., 2022b) Reduced ethylene production by ACCD production relieved salt stress in red pepper and tomato plants by inoculating *Pseudomonas* sp. and *P. azotoformans* CHB 1107, respectively (Gamalero and Glick, 2022). Another analysis revealed that halo-tolerant *Glutamicibacter* sp. and *Streptomyces* sp. GMKU 336 improve ACCD-induced salt stress mitigation in rice (Jaemsaeng et al., 2018; Ji et al., 2020).

### 8.2 Extracellular polymeric substances (EPS) production

Microorganisms synthesize a wide range of biopolymers, including polysaccharides, polyesters, and polyamides. During salinity stress, different types of microbial polysaccharides, including intracellular/extracellular polysaccharides (EPS) and exopolysaccharides (ESP), are produced, which create a boundary between cells and the surrounding environment, hence acting as a defensive measure by facilitating adhesion to biotic and abiotic surfaces, aiding in plant sustenance and alleviating desiccation stress (Ma et al., 2020). While strengthening microbial adhesion to soil particles, these polysaccharides accelerate macropore production, culminating in soil porosity and aeration, thereby initially reducing plant stress *via* soil dynamics improvement. Another feature that aids microbe survival in stressful environments is the presence of polysaccharide–lipid (PL) and lipopolysaccharide–protein (LP), a carbohydrate complex in the EPS structure (Kumar et al., 2020). Plant–microbe interaction governed by EPS establishes microenvironments that permit microorganisms to thrive in adverse circumstances and assists microbial colonization of the plant *via* enabling them to stick to root exudates. Furthermore, EPS is a slime material attached to the soil encircling plant roots *via* hydrogen bonds, cation bridges, anion adsorption mechanisms, and other mechanisms, minimizing plant exposure to hazardous ions. EPS can affect the physicochemical properties of microorganisms and limit sodium (Na^+^) uptake in salt stress. Plants inoculated with EPS-generating microbes show resilience to salinization (Chandra et al., 2021).

One such evidence has been reported by Vimal et al. (2019), where *Curtobacterium albidum* SRV4 isolated from a saline soil, when inoculated to salt-stressed paddy plants, expressed increment in EPS production along with other attributes such as nitrogen (N_2_) fixation, hydrogen cyanide (HCN), IAA, and ACC deaminase activity. *Alcaligenes* sp. AF7 promoted 263% higher EPS production in rice under salinity (300 mM NaCl) than non-saline control. Several studies proved that concurrent usage of EPS and salinity-tolerant rhizomicrobes can be effective for saline soils (Fatima et al., 2020).

### 8.3 Conglomeration of osmolytes and water homeostasis to abate osmotic stress

Hyper-osmotic stress is a salinity-related relay impact that drives plants to lose intracellular water, generating water balance disruptions. Plants undergo numerous biochemical and physiological adjustments to sustain their osmotic state, notably changes in photosynthetic parameters and osmolyte synthesis in the cytoplasm thereby maintaining cell turgor and maintaining plant metabolic processes (Ma et al., 2020). Beneficial microorganisms such as *Azospirillum, Burkholderia, Arthrobacter, Bacillus, Pseudomonas*, and *Rhizobium* also contributed to the development of osmo-protectants including proline, betaine, trehalose, glycine, phenols, and flavonoids (Hashem et al., 2019). Additionally, microbial osmo-protectants are biosynthesized quicker than those produced by their linked plants. PGPB inoculation has been proven in research to boost plant osmolyte levels. Based on several recent studies, PGPB inoculation elevates plant osmolyte expression. The enhancement could be attributed to roots absorbing microbial solutes, or PGPB could facilitate *de novo* production in plants. *Streptomyces albidoflavus* OsiLf-2, a moderately salt-tolerant (∼6% NaCl) endophytic actinomycete, synthesized significant osmolytes, such as proline, polysaccharides, and ectoine. Its inoculation elevated the proline content (by 250.3% and 49.4%) and soluble sugar content (by 20.9% and 49.4%) in rice amid salinity (Niu et al., 2022). After exposure to *B. megaterium* B26, positive modulation of plasma membrane intrinsic protein (PIP)-type plasma membrane aquaporins was observed in maize plants exposed to salt (2.59 dS m^−1^) (Marulanda et al., 2010). *A. brasilense* AZ39 inoculation increased transcription of a PIP-type aquaporin under salt environment (200 mM NaCl) (Zawoznik et al., 2011).

### 8.4 Antioxidant barricading to caulk the oxidative stress

Amplified generation of ROS including hydroxyl radical (OH^•^), single oxygen (O_2_), superoxide anion ( ${\text{O}}_{2}^{-}$ ), and hydrogen peroxide (H_2_O_2_) is prevalent in plant in a salt-stressed environment, thereby vital bio-molecules like proteins, lipids, and photosynthetic pigments incur oxidative damage together with the deterioration of cell membrane integrity *via* polysaturated fatty acid oxidation in lipid layer leading to plant death (**Table 2**). Rhizo-microbiomes combat these deleterious repercussions of ROSs in plant *via* a robust antioxidant defense system that involves secretion of enzymes like APX, CAT, SOD, and non-enzymatic low-molecular-weight antioxidant compounds including carotenoids, proline, α-tocopherol, ascorbic acid, glutathione, and β-carotenoids. Rhizospheric application of *Azotobacter* sp. in *Glycyrrhiza glabra* L., an endangered medicinally and industrially important plant, exhibited high expression of polyphenol oxidase (PPO), peroxidase (POD), and phenylalanine ammonia-lyase (PAL) and antioxidants (CAT and APX) under salinity conditions (200 mM) (Mousavi et al., 2022). *Bacillus cereus* promotes the activity of SOD, CAT, and peroxidase, thereby mitigating salt stress in *Vigna radiata* (Islam et al., 2016)*. Piriformospora indica* and *Azotobacter chrococcum* inoculation alleviates salt stress in maize and rice by upregulating the generation of non-enzymatic antioxidants carotenoids, proline, and polyphenol accumulation (Rojas-Tapias et al., 2012; Jogawat, 2019).

**Table 2:** Impact of plant growth-promoting rhizobacteria (PGPR) in the regulation of gene expressions under salt stress conditions.

Plant species	PGPR	Expressed gene	Role of gene expression under salinity stress	Reference
Maize (*Zea mays*)	*Bacillus amyloliquefacis*	*RBCS, RBCL, H*+*- PPase, HKT1, NHX1, NHX2*, and *NHX3*	Promoted seedling growth and Chl content, improved peroxidase/catalase activity and glutathione content Decreased Na^+^ toxicity	Chen et al. (2016)
	*Bacillus megaterium*	*ZmPIP*	Increased root hydraulic conductivity	Marulanda et al. (2010)
Wheat (*Triticum* *aestivum*)	*Arthrobacter protophormiae* and *B. subtilis*	*TaCTR1* and *TaDRE2*	Enhanced salt stress tolerance	Barnawal et al. (2017)
	*Dietzia natronolimnaea*	*APX, MnSOD, CAT, POD, GPX, GR, TaABARE, TaOPR1, TaMYB*, and *TaWRKY*	Increased antioxidant enzymes and proline content	Bharti et al. (2016)
Tomato (*Solanum lycopersicum*)	*Pseudomonas putida*	*Toc GTPase*	Imported chloroplast protein	Yan et al. (2014)
	*Enterobacter* sp.	*DREB2b, RD29A, RD29B, RAB18, P5CS1*, *P5CS2, MPK*, and *MPK6*	Antioxidant activity enhanced; increased proline biosynthesis	Kim et al., 2014
Arabidopsis (*Arabidopsis* sp.) and Okra (*Abelmoschus esculentus*)	*Enterobacter* sp.	*APX* and *CAT*	Increased antioxidant enzyme activities	Habib et al. (2016)
Rice *(Oryza sativa)*	*Bacillus amyloliquefaciens*	*NADPMe2, EREBP, SOS1, BADH*, and *SERK1*	Increased plant growth and salt tolerance	Nautiyal et al. (2013)

### 8.5 Salinity resilience *via* recuperation of nutritional status and ionic homeostasis

The critical element for growth and development of crops is phosphorus, facilitating storage and distribution of energy throughout photosynthesis and is required for cell division as well as the production of RNA and DNA. It is widely established that rhizo-microbiome increases the bioavailability of phosphorus to crops facing abiotic stress *via* several non-enzymatic and enzymatic microbial mechanisms including producing phosphatase, phytase enzymes, ionophores, and organic acids (Etesami, 2020). These microorganisms break down insoluble P-minerals and transform them into a solubilized state for plant to use by reducing the pH of the rhizosphere through the secretion of organic acids. Furthermore, by producing HCN, they might indirectly enhance phosphorus availability by chelating metal elements coupled with P and liberating P into the rhizosphere (Zilaie et al., 2022; Ullah et al., 2022).

Ion homeostasis is a dynamic phenomenon, and its interruption is the fundamental reason for salinity’s growth-restricting action. Sodium and potassium ion competition for molecular transporters limits entry of the latter and disrupts plant metabolic activities (Martynenko et al., 2022). Na^+^ in roots is transferred from roots to aerial regions through xylem amid high salinity, where it deposits at leaf surfaces. Consequently, excluding NaCl from plants turn out to be challenging since merely a minor fraction travel through the phloem, limiting its migration to aerial regions and producing toxicity. When Na^+^ levels rise, the Na^+^/K^+^ ratio shifts, inhibiting cytosolic operations and impacting photosynthesis and respiration together, causing the Na^+^/H^+^ antiporter (salt overlay sensitive channel 1, or SOS_1_) to efflux Na^+^. Plants induce high-affinity K^+^ transporters, which boost K^+^ import beyond Na^+^ ions, resulting in increased salt resistance (Rodriguez-Navarro and Rubio, 2006). Additionally, membrane-bound Ca^2+^ channels are activated, which is detected by SOS_3_, also known as calcineurin B-like protein (CBL4), and form complexes with CBL-interacting protein kinase; CIPK24 (also known as SOS_2_) phosphorylates SOS_1_ for its activation. This is essential for preserving the Na^+^/K^+^ ratio and thus K^+^ transporters (Gupta et al., 2022a).

Numerous growth-promoting rhizo-microorganisms aid plant ion homeostasis by lowering ion deposition in aerial portions *via* promoting Na^+^ efflux at the roots, in addition to strengthening the efficacy of K^+^ transporters, which implicitly decreases ion accumulation. Moreover, ESP synthesis by PGPR strains inhibits plant cation uptake *via* biofilm development at the root surface. During salt toxicity, PGPR (*Pseudomonas fluorescens* biotype F*, P. fluorescens* CECT 378T*, Bacillus tequilensis, B. aryabhattai, Providencia stuartii, Pantoea agglomerans*, or *Arthrobacter* sp.) inoculation culminated in an elevated K^+^/Na^+^ ratio ascribed to increased expression of ion transport regulation genes. *Pseudomonas oryzihabitans* increases the expression of the Na^+^ transporter HKT1, while also expressing the Na^+^/H^+^ antiporters NHX1 and NHX2, limiting excess Na^+^ accumulation in photosynthetic regions (Martynenko et al., 2022). Similarly, after inoculation with halo-tolerant *Glutamicibacter* sp., Na^+^ influx was prevented and K^+^ absorption was regulated in rice seedlings to preserve ionic homeostasis (Ji et al., 2020). Against salt stress, *P. indica* leveraged proton motive force produced by H^+^-ATPase at the K^+^ transporter to reduce K^+^ flow from roots and elevate K^+^ concentration in aerial parts (Yun et al., 2018).

### 8.6 Production of volatile organic compounds

Rhizospheric microorganisms can also secrete a variety of VOCs, which thus aid plant growth and development and induction of systemic resistance (ISR) to disease. VOCs are complex molecules of low molecular weight (<300 g mol−1), high vapor pressure, and low boiling point and often lipophilic in nature that modulate physiological events, and owing to their small size, they can diffuse through porous soil, liquid, and air. Propanoic acid, 5-hydroxy-methyl-furfural, butanoic acid, geosmin, camphene, β-caryophyllene, camphor, acetaldehyde, furfural, 2-methylisoborneol, α-pinene, and methanol are among the most typically released volatile biomolecules where 2-heptanol, 2-undecanone, 4-heptanone, 2-pentadecanone, 2-pentanone, and 2-tridecanone are frequently recognized as microbial VOCs. Their physical attributes enable them to serve as “info-chemicals” or “semio-chemicals” for interspecies cross-talk, primarily in non-aqueous ecosystems (Hung et al., 2013). *Trichoderma koningii* VOC treatment mitigates salt stress (100 mM NaCl) *via* promoting plant physiological defense against oxidative damage. In *Arabidopsis thaliana* exposed to *T. koningii* VOCs amid saline stress, the diminution in H2O2 content was associated by the detection of fewer necrotic cells (Jalali et al., 2017). Cappellari and Banchio (2020) demonstrated that the contribution of VOCs principally acetoin from *Bacillus amyloliquefaciens* GB03 on *Mentha piperita* in a saline environment has found the explicit increment in endogenous jasmonic acid (JA), salicylic acid, and ABA concentrations that imparts *M. piperita* growth during stress.

### 8.7 Improved photosynthesis and other physiological changes

Beneficial rhizo-microbiomes can modulate photosynthetic responses of crops under high-salt conditions. Salinity induces a series of morphological, physiological, and molecular modifications in crops like alteration in enzyme systems, impeded chlorophyll biosynthesis, malfunctioning photosynthetic apparatus, blocked electron flow from PSII to PSI, non-photochemical dissipation of heat energy, shifts in gene expression, dropped CO_2_ availability owing to hydrostatic stomatal closure, and reduction in leaf area. Salt-elicited suppression of the electron transport chain prompts pseudocyclic electron transport, promoting overdeposition of ROS, ultimately preventing plant maturity (Pan et al., 2021; Pan et al., 2022). Dysfunctional electron transport chain culminates the inhibition of electron transfer leading to electron accumulation, hence producing oxidative outburst. Furthermore, salt stress impairs photo-protection by interfering with cyclic electron transport around PSI and cytochrome *b6f*. Salinity promotes old leaves to encounter chlorosis and disintegration. The plant’s development is severely hampered because of a failure in providing required carbohydrates to young leaves (Yan et al., 2020). *P. indica* and plant growth-promoting bacteria (PGPB) substantially improved physiological responses like photosynthetic rate, stomatal conductance, transpiration, and internal CO_2_ as well as biochemical attributes such as carotenoids, chlorophylls, nitrogen, and protein content in *Trigonella foenumgraecum* (Bisht et al., 2022). The implications of salinity on photosynthesis might be short-term or long-term. Reduced CO_2_ supply, resulting from stomatal closure, is a short-term consequence of salinity, whereas salt accumulation in leaves is a long-term effect. As a result, the chlorophyll and carotenoid content falls. This decrease could potentially be attributable to increased chlorophyllase activity or altered in the lipid–protein ratio of pigment–protein complexes. During salt stress, *Methylobacterium oryzae* CBMB20 inoculation massively improved pigment contents, photosynthetic activity, antioxidant activities, and ethylene regulation-associated protein, thereby counteracting salt-induced apoptosis in rice (Roy Choudhury et al., 2022). Treatment of *P. putida* in soybean plants promoted shoot length, chlorophyll content, and plant fresh and dry weight. *Myroides* sp. JIL321 is a salt-tolerant bacterium that enhanced the chlorophyll concentration considerably in rice (Wang et al., 2022a). The photosynthetic pigment content of *P. indica* inoculated rice seedlings rose dramatically under high-salt conditions.

## 9 Molecular alterations

**Figure 5:**
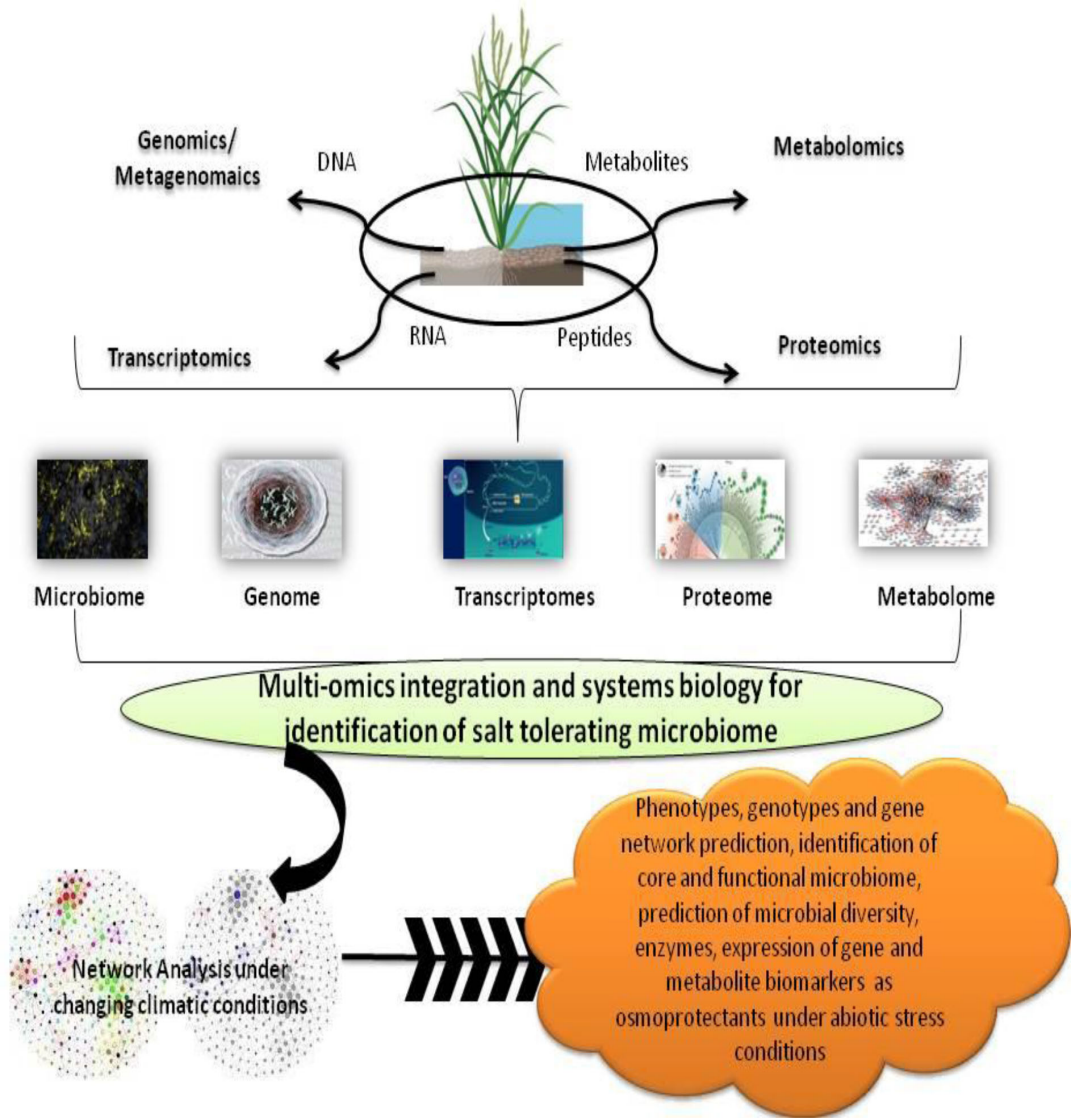
Schematic diagram showing multi-omics approaches and their beneficial impact towards improved plant health.

Despite the uncovering of a plethora of quantitative trait loci (QTLs) and genes involved with salt stress resilience in plants, our comprehension of the cellular regulatory network of salt response is still restricted. Nevertheless, with the accelerated advances in high-throughput technologies and bioinformatics during the last decade, studies on plant adaptations to salt stress have seen tremendous progress and noteworthy advances. Omics technology integrated with bioinformatics provides a new technique to research plant microorganism interactions at the molecular level (**Figure 5**). In view of the above, the transcriptomic and proteomics analysis has established itself as an effective approach in giving mechanistic paradigms of plant and microorganism relationships in recent times (Gunnaiah et al., 2012).

### 9.1 Transcription studies

A transcriptomic-based genome-wide gene expression analysis is an excellent method for analyzing the gene regulation networks of complex plant traits. Transcriptomics is categorized into two types: cDNA microarray-based hybridization and RNA-seq, which leverages next-generation sequencing (NGS). The purpose of a cDNA microarray is to fix a gene probe on a stationary layer instrument using micromachining technology and then hybridize with the labeled sample for evaluation by measuring the hybridization signal. However, owing to the predesigned templates, cDNA microarray could not identify new transcripts and the outcomes were erroneous when gene expression was either low or too high (Dai et al., 2022). RNA-seq, on the other hand, is a high-throughput sequencing approach that may not merely represent mRNA, small RNA, and non-coding RNA expression patterns, yet also finds novel transcripts or even a novel gene and is consequently more often employed than cDNA microarray currently. A transcriptomic study of salt-stressed cotton plant inoculated with *B. subtilis* and *B. pumilus* exhibited 556 (481 upregulated and 75 downregulated) and 943 (536 upregulated and 407 downregulated) differentially expressed genes (DEGs), respectively. Its KEGG testing showed that plants treated with *B. pumilus* distinctively expressed ascorbate and aldarate metabolism pathways, as well as glyoxylate and dicarboxylate metabolism pathways, and plants treated with *B. subtilis* uniquely expressed pentose and glucuronate interconversions pathway genes, in addition to plant pathogen interaction and phyto-hormone signaling pathway genes, which are expressed in both (Akbar et al., 2022). Analogously, transcriptome analysis in *Arabidopsis thaliana* validated that Lys-motif receptor LYK4 induced by *Enterobacter* sp. SA187 regulates salt tolerance (Rolli et al., 2022). Rabbani et al. (2003) employed a microarray to identify 57 high salt-induced and 43 ABA-induced genes, and 39 genes were expressed by both, indicating a considerable inter-modulation among salt stress and ABA stress signaling pathways.

Small RNAs (20–40 nucleotides in length) including endogenous short interfering RNAs (siRNAs) and microRNAs (miRNAs) contribute significantly to phyto-stress response-mediated gene silencing at the transcriptional and posttranscriptional levels, together with the control of plant growth and development (Dai et al., 2022). Tripathi et al. (2018) reported NGS to evaluate the expression of miRNAs in salt-tolerant rice with glyoxal glycosidase overexpression and discovered numerous salt-mediated miRNAs regulating salinity resilience. Parmar et al. (2020) applied high-throughput sequencing to identify multiple differentially expressed miRNAs sensitive to salt stress.

### 9.2 Proteomic studies

Although the majority of stress-responsive genes are controlled at the transcriptional level, variations at the transcriptional level are not necessarily complemented by shifts in protein abundance attributed to translation and posttranslational regulation. Furthermore, protein is a fundamental function operator. As a result, proteome analysis is pivotal and proteomics is being established on this foundation. Proteomics is the high-throughput investigation of protein parameters like protein expression, posttranslational modification, and protein–protein interaction, together with protein localization within the cells and tissues. Quantitative proteomics is centered on mass spectrometry, which is a lucrative technique for exploring the intricate dynamics of plant salt stress response (Dai et al., 2022; Gupta et al., 2022b).

Prabhukarthikeyan et al. (2022) validated that inoculation of *B. megaterium* strain BS11 upregulated proteins associated with plant metabolism, transcription, transporter, signaling, defense and stress responses in rice plants using proteomic analysis. Proteome profiling of field soil supplied with *B. subtilis* CP4 and Arbuscular mycorrhizal fungi (AMF) microbial consortia divulged the promotion of differentially abundant proteins (DAP) including histones, glutenin, avenin, and ATP synthase in abiotic stressed wheat, resulting in better yield and biofortification (Yadav et al., 2022). An integrated proteomics and transcriptomics study successfully identified 64 DAP participating in “carbon fixation in photosynthetic organisms” as well as few amino acid metabolism pathways that may be related to C and N allocations and AMF-mediated *Suaeda salsa* adaptation in moderate saline conditions (100 mM NaCl) (Diao et al., 2022). Similarly, proteomics data demonstrated that treatment of wheat seedlings with *Bacillus* sp. wp-6 during salt stress (150 mM NaCl) resulted in the expression of 88 DAP, the top 10 of which were associated with valine, leucine and isoleucine degradation, ribosome, peroxisome, metabolic pathways, glyoxylate and dicarboxylate metabolism, galactose metabolism, fatty acid metabolism, fatty acid degradation, amino sugar and nucleotide sugar metabolism, and alpha-linolenic acid metabolism (Zhao et al., 2022). Shotgun proteomics showed upregulation of several key proteins such as photosystem I *psaK*, Rubisco subunits, glyceraldehyde-3-phosphate dehydrogenase, succinate dehydrogenase and glycine decarboxylase, and salt stress response proteins such as CAT and glutathione S-transferase (antioxidants), proline-rich precursor protein (osmolyte), and NADP-dependent malic enzyme (ABA signaling), which elicited stress tolerance in soybean by co-inoculation of *Rhizobium* sp. SL42, *Hydrogenophaga* sp. SL48, and *Bradyrhizobium japonicum* 532C (Ilangumaran et al., 2022).

## 10 Constraints and future trends

Despite the positive laboratory achievements for microbes and microbe-based products, they still have not made it to the field as they ought to, which makes stakeholders and environmentalists more anxious. This failure was caused by a number of factors, which are discussed in this section.

### 10.1 Plant selectivity of microorganisms

The primary obstacle with implementing PGPMs is their erratic response in the field than in regulated conditions. Although efficient experiments are completed in laboratory, predicting how organisms will behave in a natural habitat is challenging. The primary factor for this is plant–microbe specificity. The viability of the desired inoculants and root colonization, meanwhile, are also crucial considerations for PGPR producers (Lakshmi and Usha, 2022). A certain population density at the colonization region on the host plant must be attained by the inoculants for effective response. However, root colonization is unaffected by the PGPR’s crop specificity (Mcnear, 2013; Mushtaq, 2020). Other factors having a significant impact on the viability and root colonization of PGPM are environment parameters such soil type, temperature, moisture, and the prevalence of other competing rhizo-microbiota, which differ depending on the crops and the field type. This renders it difficult for PGPR manufacturers to design PGPR inoculants for different crops that perform efficiently in diverse environmental conditions (Saharan and Nehra, 2011). To address these issues and inconsistencies relating to PGPR’s adaptability, survivability, colonization, and specificity, microbial consortia may offer a more effective solution than a single microbe. A well-formulated microbial consortium includes two or more compatible microbial species from distinct genera or phyla with synergistically interacting properties and varying adaptation to various environmental factors. Additionally, they indirectly support the host plant by secreting substances that encourage the colonization of other beneficial rhizospheric phyto-microbiota or by producing specific signaling compounds that further facilitate plant–microbe interaction. Unfortunately, finding a compatible microbial partner might be troublesome (Naamala and Smith, 2020). Additionally, certain microbes can prevent the growth of others by secreting a toxic chemical or impair their capacity to stimulate crop growth. Regarding the challenges and uncertainties imposed by using microbial strains, competent microbe-based substitutes that promote plant growth in adverse environmental conditions must be explored. Thuricin 17 and lipo-chitooligosaccharides, two microbial compounds, positively influenced plant growth amid high saline and drought stress (Shah et al., 2021). Nevertheless, micro-based products have the potential to be more effective than microbial strains for different crop varieties under different environmental conditions. Future molecular studies, however, are necessary to comprehend how microbial inoculants interact with plants and their prolonged impact on host rhizosphere chemistry.

### 10.2 Legal and regulatory issues

**Figure 6:**
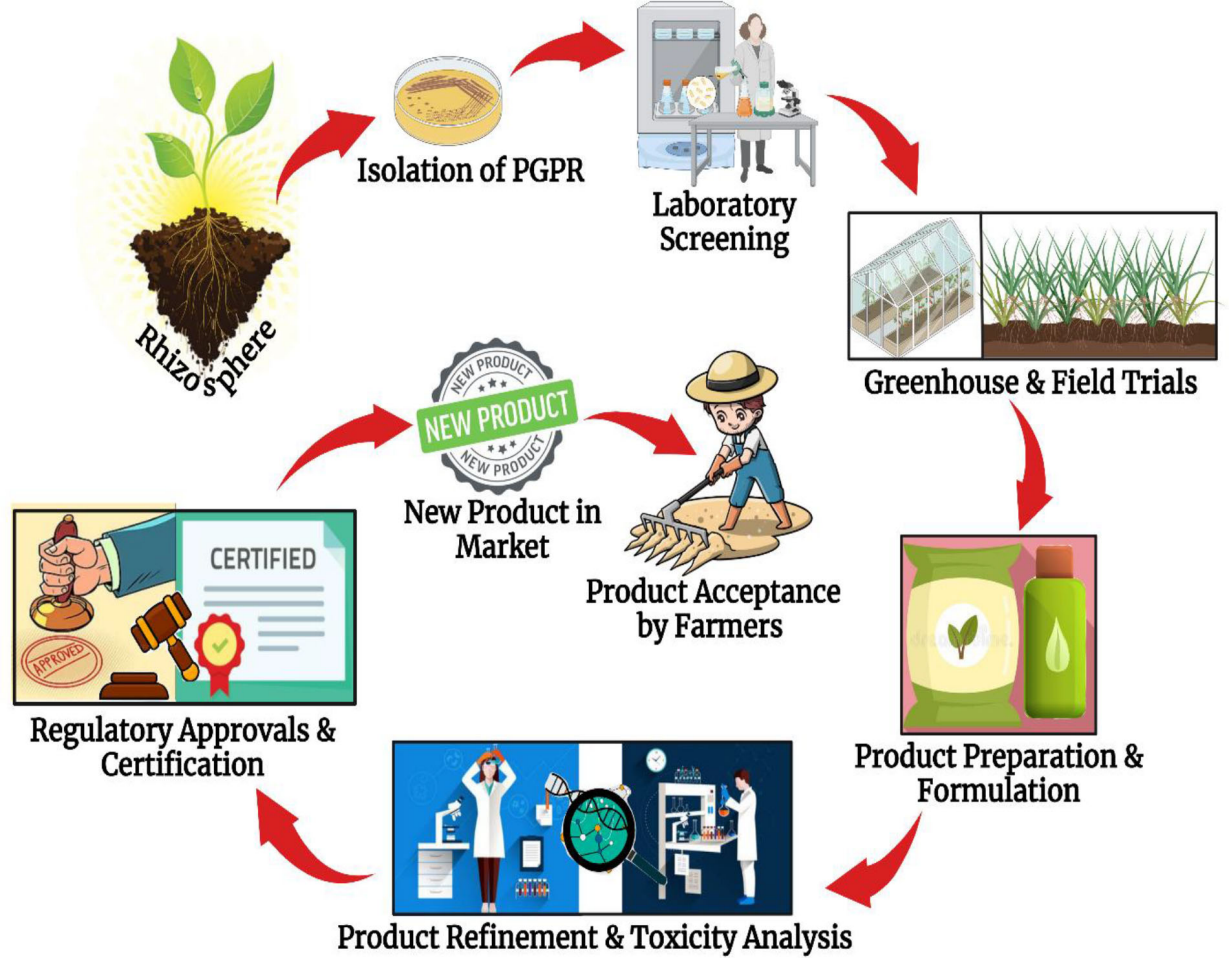
Blueprint of commercialization of a new PGPR product to be administered on the field by farmers and crop producers.

To prevent the manufacturing and dissemination of lethal or toxic microorganisms, each country and occasionally subnational jurisdictions have established rather stringent risk assessments and testing criteria for any microbe-based product that is intended to be applied in the field. The road map of commercialization of a PGPR-based product is illustrated in **Figure 6**. The entire registration procedure for microbe-based products is time-consuming, involves complicated documentation, and is overpriced. The fact that registration and regulatory policies differ by country/nation owing to particular regulations and norms, when the products are required to be registered across several countries, further complicates this already complicated procedure. As a result, the product becomes practically region-specific (Tabassum et al., 2017). The Directorate General of Health or another relevant regulatory body of a nation must approve and certify a product, which is often required for product registration. When the tests are successful, the manufacturers will be given a certification approving the legitimate manufacturing and commercialization of the product in compliance with strict regulations and guidelines set out by certification authorities. Agricultural and food sustainability already suffers tremendously from the constantly changing climate; thus, the technologies that could assist the agroecosystem sustenance should have less complex registration and regulatory standards (Backer et al., 2018). Moreover, countries have to be more adaptable and develop less complicated legislation for the import, transport, and registration of micro-based technologies. This could make it easier for firms and industries to expand and market their stress-alleviating PGPR products both inside and outside of the country of origin.

### 10.3 Farmers’ outlook

The Green Revolution has fostered the overexploitation of agrochemicals and other agricultural approaches. Because of their effectiveness and quick results, farmers and agricultural producers gained a profound sense of trust that makes it challenging to adopt or use other novel technologies or alternatives by them (Moser et al., 2008). Their undeniable contribution to agriculture is indispensable; however, their detrimental contribution to GHG emissions from agricultural practices is a serious worldwide problem that has slightly eroded farmers’ faith in them. Although no technology has yet been able to completely replace agrochemicals, some sustainability initiatives, such as the use of organic fertilizers, humic substances, or bio-based products (compounds made from microorganisms and their products), have paved the way for their entry into this system while also aiding to slow down the consequences of climate change by lessening the consumption of agro-chemicals to a certain extent (Shah et al., 2021). Farmers’ mindset and their capacity for risk-taking are the main regulatory body that permits these innovative technologies to reach fields, especially microbe-based goods, which also comes with uncertainties. Farmers’ primary dilemma with microbial technologies is that there is no sufficient evidence of their efficacy in the field. It might be challenging for farmers to switch to a new technology with uncertainties while abandoning the ones (chemicals and fertilizers) that have provided them with high agricultural production for the last 50 years. Another issue is that agrochemicals are more successful in small quantities for a large area, showing high effectiveness towards pathogen control and nutrient availability than PGR products. In comparison to PGPR products, agrochemicals are significantly less costly. Since PGPR products are relatively new to the market, their price will not fall until they are widely distributed and mass manufactured. Additionally, PGPR products have a lower “cost-to-benefit ratio” than chemical supplements (Ali et al., 2021). These shortcomings prevent farmers from adopting PGPR products broadly. To eradicate these issues and discrepancies, a number of actions must be implemented to train farmers about global climate change and its detrimental implications, along with the contribution of PGPR products in sustainable agriculture with a view to protect the environment. It is crucial to conduct scientific research to lower the price of PGPR products, increase the physical proof of their efficacy, and address the specific requirements of farmers.

### 10.4 Modern tools

Considering the underlying knowledge of how plants and microbes interact, indigenous rhizospheric microbial diversity will be exploited significantly in agricultural fields to enhance crop viability against changing climatic factors. There is no evidence, but it is likely that the PGPR inoculants operate differently from other stress-reduction agents to alleviate abiotic stresses (salinity and drought). To thoroughly comprehend the correlations and variations between microbe-elicited resilience to salt and drought adaptability, more research is required. This research should focus on the molecular mechanisms by which microorganisms support themselves and their symbiotic partner, as well as on innovations that imitate natural microbial interactions under abiotic stressed conditions.

Crop genome editing is now possible thanks to the commencement of clustered regularly interspaced short palindromic repeat (CRISPR) technology, proteomics, transcriptomics, and metabolomics. They offer a number of benefits, namely, convenience, adaptability, excellent performance, and the capacity to operate with different targets at once. Additionally, it makes it possible to have adequate information of the signaling agents participating in plant–microbe interactions, to decipher genomics and metabolomics, and to precisely modify genetic information for increased agricultural productivity amid stress (Chaudhary and Shukla, 2019; Marotkar et al., 2020). The establishment of stress resistance varieties by breeding and genetic engineering is a critical yet prolonged method, whereas salt-tolerating PGPR inoculants relieving abiotic stressors in the crop opens up a revolutionary approach for employing PGPRs in climate smart agriculture systems.

Synthetic microbial communities (SynComs) have attracted a lot of attention lately. SynComs are small consortia of microbes constructed to somewhat replicate the structure and function of the microbiome as seen in its natural environment. In order to preserve some of the natural interactions between microorganisms and hosts while also reducing the complexity of the microbial community, a number of benefits that cannot be performed by a single microbe are provided (De Souza et al., 2020).

Artificial intelligence (AI) is a subfield of computer science that employs tools and algorithms to endeavor to imitate human cognitive abilities. AI algorithms are capable of self-learning without expecting parental distribution. The goal of AI is to build a prediction model that explores undiscovered commonalities in a challenging conundrum (Anitha Mary et al., 2022). In relation to this, machine learning (ML), a subset of AI, gives computers the capacity to train without deliberate programming by utilizing statistical techniques to permit machines to get better over time. As a corollary, AI and ML efficiently automate the construction of analytical models and enable machines to autonomously produce novel situations (Wang et al., 2022b). Examples of such broadly used models and algorithms include (i) the Artificial Neural Network (ANN, used in nearly all AI-based application domains in real-life data), (ii) the Support Vector Machine (SVM, first created for linear categorization tasks), (iii) the Nonlinear Support Vector Regression (NLSVR), and (iv) Random Forest (RF). Numerous agricultural and environmental disciplines, such as plant-based, pedological, and salinity-based studies, have effectively used the AI and ML algorithms. However, because soil salinization is frequently a very complicated and nonlinear variable, data processing using AI and ML techniques may produce better results than traditional statistical methods in the classification and prediction of soil salinity (Ondrasek et al., 2022). The difficulties in evaluating and modeling soil salinity have been addressed in a small number of research using various statistical and AI algorithms, for instance, the use of an SVM classifier model with inputs for multispectral and textural features to classify salinity-influenced soils. Consequently, when modeling soil salinity, SVM was successful in extracting salinization and soil-thematic details from the inputs, resulting in a reliable classification score (Cai et al., 2010.

## 11 Conclusion

Soil salinity causes severe crop loss worldwide as a result of threatening global food security. Under climate change-induced salt stress, plants activate various pathways controlling ROS scavenging, expression of stress-responsive genes, and protein and cellular membrane stability, which become lethal amid prolonged stressors. The rhizo-microbiome aligned with crops constitutes a substantial element of ecosystems and is essential for giving plants the capacity to evolve in response to climatic conditions (for instance, drought and salinity). Considering this situation, rhizospheric microbiota can save agriculture crops through the production of phytohormone, EPS, ESP, osmolytes, and ACCD; maintenance of ionic homeostasis; reduction of osmotic and oxidative stress; and modulation of antioxidative defense mechanisms. These actions can alleviate the detrimental implications of abiotic stressors while also promoting plant growth and productivity. Researchers have well-formulated several PGPRs and PGPR-based products exhibiting great results on fields. Moreover, omics technologies have been promising in uncovering the stress-induced cellular regulatory network. Concentrated studies are required to identify the indigenous microbial inoculants, resolve the quandary of application strategies, and carry out field trials to establish sustaining agriculture development under climatic stress conditions.

## Author contributions

KK, BS, and SN conceived and planned the overall idea as well as wrote the review manuscript. KK, SN, BS, AK, ST, and RM critically reviewed the manuscript. All authors read and approved the submitted final version of the manuscript.

## References

[B1] Abd El-Ghany T. M. Masrahi Y. S. Mohamed A. Abboud A. Alawlaqi M. M. Elhussieny N. I. (2015). Maize (*Zea mays* l.) growth and metabolic dynamics with plant growth promoting rhizobacteria under salt stresses. J. Plant Pathol. Microb. 6, 305. doi: 10.4172/2157-7471.1000305

[B2] Abulfaraj A. A. Jalal R. S. (2021). Use of plant growth promoting bacteria to enhance salinity stress in soybean (*Glycine max* l.) plants. Saudi J. Biol. Sci. 28, 3823–3834. doi: 10.1016/j.sjbs.2021.03.053 34220237 PMC8241701

[B3] Ahanger M. A. Agarwal R. M. (2017). Salinity stress induced alterations in antioxidant metabolism and nitrogen assimilation in wheat (*Triticum aestivum* l.) as influenced by potassium supplementation. Plant Physiol. Biochem. 115, 449–460. doi: 10.1016/j.plaphy.2017.04.017 28478373

[B4] Akbar A. Han B. Khan A. H. Feng C. Ullah A. Khan A. S.. (2022). A trans-criptomic study reveals salt stress alleviation in cotton plants upon salt tolerant PGPR inoculation. Environ. Exper. Bot. 200, 104928. doi: 10.1016/j.envexpbot.2022.104928

[B5] Ali B. M. Ang F. van der Fels-Klerx H. J. (2021). Consumer willingness to pay for plant based foods produced using microbial applications to replace synthetic chemical inputs. PloS One 16 (12), e0260488. doi: 10.1371/journal.pone.0260488 34874958 PMC8651115

[B6] Ali B. Wang X. Saleem M. H. Sumaira Hafeez A. Afridi M. S.. (2022). PGPR mediated salt tolerance in maize by modulating plant physiology, antioxidant defense, compatible solutes accumulation and bio-surfactant producing genes. Plants 11 (3), 345. doi: 10.3390/plants11030345 35161325 PMC8840115

[B7] Allison S. D. Martiny J. B. H. (2008). Resistance, resilience and redundancyin microbial communities. PNAS 105 (1), 11512–11519. doi: 10.1073/pnas.0801925105 18695234 PMC2556421

[B8] Allotey D. F. K. Asiamah R. D. Dedzoe C. D. Nyamekye A. L. (2008). Physico-chemical properties of three salt affected soils in the lower Volta basin and management strategies for their sustainable utilization. West Afr. J. Appl. Ecol. 12 (1), 1–14. doi: 10.4314/wajae.v12i1.45776

[B9] Anitha Mary X. Popov V. Raimond K. Johnson I. Vijay S. J. (2022). “Scope and recent trends of artificial intelligence in Indian agriculture,” in The digital agricultural revolution: Innovations and challenges in agriculture through technology disruptions, 1–24. Wiley Online Library. doi: 10.1002/9781119823469.ch1

[B10] Ansari F. Jabeen M. Ahmad I. (2021). *Pseudomonas azotoformans* FAP5, a novel biofilm forming PGPR strain, alleviates drought stress in wheat plant. Int. J. Environ. Sci. Technol. 18, 1–16. doi: 10.1007/s13762-020-03045-9

[B11] Ansari M. Shekari F. Mohammadi M. H. Juhos K. Végvári G. Biró B. (2019). Salt tolerant plant growth promoting bacteria enhanced salinity tolerance of salt tolerant alfalfa (*Medicago sativa* l.) cultivars at high salinity. Acta Physiol. Plant 41, 195. doi: 10.1007/s11738-019-2988-5

[B12] Asaf S. Hamayun M. Khan A. L. Waqas M. Khan M. A. Jan R. (2018). Salt tolerance of *Glycine max.* l induced by endophytic fungus *Aspergillus flavus* CSH1, *via* regulating its endogenous hormones and anti-oxidative system. Plant Physiol. Biochem. 128, 13–23. doi: 10.1016/j.plaphy.2018.05.007 29751251

[B13] Ayaz M. Ali Q. Farzand A. Khan A. R. Ling H. Gao X. (2021). Nematicidal volatiles from *Bacillus atrophaeus* GBSC56 promote growth and stimulate induced systemic resistance in tomato against meloidogyne incognita. Int. J. Mol. Sci. 22, 5049. doi: 10.3390/ijms22095049 34068779 PMC8126219

[B14] Backer R. Rokem J. S. Ilangumaran G. Lamont J. Praslickova D. Ricci E. (2018). Plant growth promoting rhizobacteria: context, mechanisms of action and roadmap to commercialization of bio-stimulants for sustainable agriculture. Front. Plant Sci. 9, 1473. doi: 10.3389/fpls.2018.01473 30405652 PMC6206271

[B15] Bal H. B. Nayak L. Das S. Adhya T. K. (2013). Isolation of ACC deaminase producing PGPR from rice rhizosphere and evaluating their plant growth promoting activity under salt stress. Plant Soil 366 (1), 93–105. doi: 10.1007/s11104-012-1402-5

[B16] Banerjee A. Bareh D. A. Joshi S. R. (2017). Native micro-organisms as potent bio-inoculants for plant growth promotion in shifting agriculture systems. J. Soil Sci. Plant Nutr. 17, 127–140. doi: 10.4067/S0718-95162017005000010

[B17] Bardgett R. D. Freeman C. Ostle N. J. (2008). Microbial contributions to climate change through carbon cycle feedbacks. ISME J. 2, 805–814. doi: 10.1038/ismej.2008.58 18615117

[B18] Barnawal D. Bharti N. Pandey S. S. Pandey A. Chanotiya C. S. Kalra A. (2017). Plant growth promoting rhizobacteria enhances wheat salt and drought stress tolerance by altering endogenous phytohormone levels and TaCTR1/TaDREB2 expression. Physiol. Plant 161 (4), 502–514. doi: 10.1111/ppl.12614 28786221

[B19] Belimov A. A. Zinovkina N. Y. Safronova V. I. Litvinsky V. A. Nosikov V. V. Zavalin A. A. (2019). Rhizobial ACC deaminase contributes to efficient symbiosis with pea (*Pisum sativum* l.) under single and combined cadmium and water deficit stress. Environ. Experi. Bot. 167, 103859. doi: 10.1016/j.envexpbot.2019.103859

[B20] Berlanas C. Berbegal M. Elena G. Laidani M. Cibriain J. F. Sagues A. (2019). The fungal and bacterial rhizosphere microbiome associated with grapevine rootstock genotypes in mature and young vineyards. Front. Microbiol. 10, 1142. doi: 10.3389/fmicb.2019.01142 31178845 PMC6538693

[B21] Bharti N. Pandey S. S. Barnawal D. Patel V. K. Kalra A. (2016). Plant growth promoting rhizobacteria *Dietzia natronolimnaea* modulates the expression of stress responsive genes providing protection of wheat from salinity stress. Sci. Rep. 6, 34768. doi: 10.1038/srep34768 27708387 PMC5052518

[B22] Bhat M. A. Kumar V. Bhat M. A. Wani I. A. Dar F. L. Farooq I.. (2020). Mechanistic insights of the interaction of plant growth promoting rhizobacteria (PGPR) with plant roots toward enhancing plant productivity by alleviating salinity stress. Front. Microbiol. 11, 1952. doi: 10.3389/fmicb.2020.01952 32973708 PMC7468593

[B23] Bisht S. Singh S. Singh M. Sharma J. G. (2022). Augmentative role of *Piriformospora indica* fungus and plant growth promoting bacteria in mitigating salinity stress in *Trigonella foenumgraecum* . J. Appl. Biol. Biotechnol. 10 (1), 85–94. doi: 10.7324/JABB.2021.100111

[B24] Bowyer C. Withana S. Fenn I. Bassi S. Lewis M. Cooper T.. (2009). Land degradation and desertification policy department economic and scientific policy. IP. IP/A/ENVI/ST, 102.

[B25] Cai S. Zhang R. Liu L. Zhou D. (2010). A method of salt-affected soil information extraction based on a support vector machine with texture features. Math. Comput. Model. 51 (11-12), 1319–1325. doi: 10.1016/j.mcm.2009.10.037

[B26] Cappellari L. D. R. Banchio E. (2020). Microbial volatile organic compounds produced by *Bacillus amyloliquefaciens* (GB03) ameliorate the effects of salt stress in *Mentha piperita* principally through acetoin emission. J. Plant Growth Reg. 39 (2), 764–775. doi: 10.1007/s00344-019-10020-3

[B27] Chandra P. Singh A. Choudhary M. Yadav R. K. (2021). “Role of plant growth promoting rhizobacteria (PGPR) in mitigating salt stress,” in Agriculturally important microorganisms (CRC Press), 65–90.

[B28] Chatterjee P. Samaddar S. Niinemets U. Sa T. M. (2018). *Brevibacterium linens* RS16 confers salt tolerance to *Oryza sativa* genotypes by regulating antioxidant defense and h^+^ ATPase activity. Microbiol. Res. 215, 89–101. doi: 10.1016/j.micres.2018.06.007 30172313

[B29] Chaudhary T. Shukla P. (2019). Bio-inoculant capability enhancement through metabolomics and systems biology approaches. Briefings Funct. Genomics 18 (3), 159–168. doi: 10.1093/bfgp/elz011 31232454

[B30] Chen L. Liu Y. Wu G. Veronican Njeri K. Shen Q. Zhang N.. (2016). Induced maize salt tolerance by rhizosphere inoculation of *Bacillus amyloliquefaciens* SQR9. Physiol. Plant 158 (1), 34–44. doi: 10.1111/ppl.12441 26932244

[B31] Chinnaswamy A. Coba de la Peña T. Stoll A. de la Peña R. D. Bravo J.. (2018). A nodule endophytic *Bacillus megaterium* strain isolated from *Medicago polymorpha* enhances growth, promotes nodulation by *Ensifer medicae* and alleviates salt stress in alfalfa plants. Ann. Appl. Biol. 172 (3), 295–308. doi: 10.1111/aab.12420

[B32] Dai L. Li P. Li Q. Leng Y. Zeng D. Qian Q. (2022). Integrated multi-omics perspective to strengthen the understanding of salt tolerance in rice. Inter. J. Mole. Sci. 23 (9), 5236. doi: 10.3390/ijms23095236 PMC9105537 35563627

[B33] Debnath S. Chandel R. K. Devi K. Khan Z. (2021). “Mechanism and molecular response of induced genotoxicity and oxidative stress in plants,” in Induced genotoxicity and oxidative stress in plants (Singapore: Springer), 213–227.

[B34] del Carmen Orozco-Mosqueda M. Glick B. R. Santoyo G. (2020). ACC deaminase in plant growth-promoting bacteria (PGPB): an efficient mechanism to counter salt stress in crops. Microbiol. Res. 235, 126439. doi: 10.1016/j.micres.2020.126439 32097862

[B35] Desoky E. S. M. Saad A. M. El-Saadony M. T. Merwad A. R. M. Rady M. M. (2020). Plant growth promoting rhizobacteria: Potential improvement in antioxidant defense system and suppression of oxidative stress for alleviating salinity stress in *Triticum aestivum* (L.) plants. Biocat. Agric. Biotechnol. 30, 101878. doi: 10.1016/j.bcab.2020.101878

[B36] De Souza R. S. C. Armanhi J. S. L. Arruda P. (2020). From microbiome to traits: designing synthetic microbial communities for improved crop resiliency. Front. Plant Sci. 11, 1179. doi: 10.3389/fpls.2020.01179 32983187 PMC7484511

[B37] Diao F. Jia B. Wang X. Luo J. Hou Y. Li F. Y.. (2022). Proteomic analysis revealed modulations of carbon and nitrogen by arbuscular mycorrhizal fungi associated with the halophyte *Suaeda salsa* in a moderately saline environment. Land Degrad. Dev. 33 (11), 1933–1943. doi: 10.1002/ldr.4274

[B38] Dietz K. J. Jacquot J. P. Harris G. (2010). Hubs and bottlenecks in plant molecular signaling networks. New Phytol. 188, 919–938. doi: 10.1111/j.1469-8137.2010.03502.x 20958306

[B39] Eggermont H. Balian E. Azevedo J. M. N. Beumer V. Brodin T. Claudet J.. (2015). Nature-based solutions: New influence for environmental management and research in Europe. GAIA 24 (4), 243–248. doi: 10.14512/gaia.24.4.9

[B40] Elkelish A. A. Soliman M. H. Alhaithloul H. A. El-Esawi M. A. (2019). Selenium protects wheat seedlings against salt stress mediated oxidative damage by up-regulating antioxidants and osmolytes metabolism. Plant Physiol. Biochem. 137, 144–153. doi: 10.1016/j.plaphy.2019.02.004 30784986

[B41] Etesami H. (2018). Can interaction between silicon and plant growth promoting rhizobacteria benefit in alleviating abiotic and biotic stresses in crop plants? Agric. Ecosyst. Environ. 253, 98–112. doi: 10.1016/j.agee.2017.11.007

[B42] Etesami H.. (2020). Plant–microbe interactions in plants and stress tolerance. Plant Life Under Changing Environ. 2020, 355–395. doi: 10.1016/B978-0-12-818204-8.00018-7

[B43] Fahad S. Hussain S. Matloob A. Khan F. A. Khaliq A. Saud S.. (2015). Phyto-hormones and plant responses to salinity stress: a review. Plant Growth Reg. 75 (2), 391–404. doi: 10.1007/s10725-014-0013-y

[B44] FAO (1988). UNESCO Soil map of the world, revised legend, with corrections and updates. World Soil Res. Rep., 60–140.

[B45] FAO (2002). Gender and sustainable development in dry lands: an analysis of field experiences. original and complete version of the present document (Rome: FAO).

[B46] Fathalla Abd el-mageed A. A. (2020). Salt tolerance enhancement of wheat (*Triticum asativium* l.) genotypes by selected plant growth promoting bacteria. AIMS Microbiol. 6 (3), 250–271. doi: 10.3934/microbiol.2020016 33134743 PMC7595838

[B47] Fatima T. Mishra I. Verma R. Arora N. K. (2020). Mechanisms of halo-tolerant plant growth promoting alcaligenes sp. involved in salt tolerance and enhancement of the growth of rice under salinity stress. 3 Biotech. 10 (8), 1–12. doi: 10.1007/s13205-020-02348-5 32832323 PMC7392994

[B48] Fazal A. Bano A. (2016). Role of plant growth promoting rhizobacteria (PGPR), biochar and chemical fertilizer under salinity stress. Commun. Soil Sci. Plant Anal. 47 (17), 1985–1993. doi: 10.1080/00103624.2016.1216562

[B49] Gamalero E. Glick B. R. (2022). Recent advances in bacterial amelioration of plant drought and salt stress. Biol 11 (3), 437. doi: 10.3390/biology11030437 PMC8945159 35336811

[B50] Garcia-Caparros P. De Filippis L. Gul A. Hasanuzzaman M. Ozturk M. Altay V.. (2021). Oxidative stress and antioxidant metabolism under adverse environmental conditions: a review. Botanical Rev. 87 (4), 421–466. doi: 10.1007/s12229-020-09231-1

[B51] Golldack D. Li C. Mohan H. Probst N. (2013). Gibberellins and abscisic acid signal crosstalk: living and developing under unfavorable conditions. Plant Cell Rep. 32 (7), 1007–1016. doi: 10.1007/s00299-013-1409-2 23525744

[B52] Gou W. Tian L. Ruan Z. Zheng P. Chen F. Zhang L. (2015). Accumulation of choline and glycinebetaine and drought stress tolerance induced in maize (*Zea mays*) by three plant growth promoting rhizobacteria (PGPR) strains. Pak. J. Bot. 47, 581–586.

[B53] Gunnaiah R. Kushalappa A. C. Duggavathi R. Fox S. Somers D. J. (2012). Integrated metaboloproteomic approach to decipher the mechanisms by which wheat QTL (Fhb1) contributes to resistance against *Fusarium graminearum* . PloS One 7, e40695. doi: 10.1371/journal.pone.0040695 22866179 PMC3398977

[B54] Gupta A. Bano A. Rai S. Mishra R. Singh M. Sharma S.. (2022a). Mechanistic insights of plant-microbe interaction towards drought and salinity stress in plants for enhancing the agriculture productivity. Plant Stress 4, 100073. doi: 10.1016/j.stress.2022.100073

[B55] Gupta A. Mishra R. Rai S. Bano A. Pathak N. Fujita M.. (2022b). Mechanistic insights of plant growth promoting bacteria mediated drought and salt stress tolerance in plants for sustainable agriculture. Intern. J. Mole. Scien. 23 (7), 3741. doi: 10.3390/ijms23073741 PMC8998651 35409104

[B56] Gupta A. Rai S. Bano A. Khanam A. Sharma S. Pathak N. (2021). Comparative evaluation of different salt tolerant plant growth promoting bacterial isolates in mitigating the induced adverse effect of salinity in *pisum sativum* . Bioint. Res. Appl. Chem. 11 (5), 13141–13154. doi: 10.33263/BRIAC115.1314113154

[B57] Habib S. H. Kausar H. Saud H. M. (2016). Plant growth promoting rhizobacteria enhance salinity stress tolerance in okra through ROS scavenging enzymes. BioMed. Res. Int. doi: 10.1155/2016/6284547 PMC4756578 26951880

[B58] Hamlet I. Muller V. (2013). Molecular mechanisms of adaptation of the moderately halophilic bacterium thiobacilli’s halophiles to its environment. Life 3, 234–243. doi: 10.3390/life3010234 25371341 PMC4187189

[B59] Haroon U. Liaquat F. Khizar M. Akbar M. Saleem H. Arif S.. (2021). Isolation of halo-tolerant bacteria from rhizosphere of khewra salt mine halophytes and their application to induce salt tolerance in wheat. Geomicrobiol. J. 38 (9), 768–775. doi: 10.1080/01490451.2021.1946624

[B60] Hashem H. A. Mansour H. A. El-Khawas S. A. Hassanein R. A. (2019). The potentiality of marine macro-algae as bio-fertilizers to improve the productivity and salt stress tolerance of canola (*Brassica napus* l.) plants. Agron 9 (3), 146. doi: 10.3390/agronomy9030146

[B61] Hassani A. Azapagic A. Shokri N. (2020). Predicting long term dynamics of soil salinity and sodicity on a global scale. Proceed. Nat. Acad. Sci. 117 (52), 33017–33027. doi: 10.1073/pnas.2013771117 PMC7776813 33318212

[B62] He Z. He C. Zhang Z. Zou Z. Wang H. (2007). Changes of anti-oxidative enzymes and cell membrane osmosis in tomato colonized by arbuscular mycorrhizae under NaCl stress. Colloids Surf B Biointerfaces 59 (2), 128–133. doi: 10.1016/j.colsurfb.2007.04.023 17560092

[B63] Ho A. Lonardo D. P. D. Bodelier P. L. E. (2017). Revisiting life strategy concepts in environmental microbial ecology. FEMS Microbiol. Ecol. 93, fix006. doi: 10.1093/femsec/fix006 28115400

[B64] Hung R. Lee S. Bennett J. W. (2013). *Arabidopsis thaliana* as a model system for testing the effect of *Trichoderma* volatile organic compounds. Fungal Ecol. 6 (1), 19–26. doi: 10.1016/j.funeco.2012.09.005

[B65] Igiehon N. O. Babalola O. O. Cheseto X. Torto B. (2021). Effects of rhizobia and arbuscular mycorrhizal fungi on yield, size distribution and fatty acid of soybean seeds grown under drought stress. Microbiol. Res. 242, 126640. doi: 10.1016/j.micres.2020.126640 33223380

[B66] Ilangumaran G. Smith D. L. (2017). Plant growth promoting rhizobacteria in amelioration of salinity stress: a systems biology perspective. Fron. Plant Sci. 8, 1768. doi: 10.3389/fpls.2017.01768 PMC5660262 29109733

[B67] Ilangumaran G. Subramanian S. Smith D. L. (2022). Soybean leaf proteomic profile influenced by rhizobacteria under optimal and salt stress conditions. Front. Plant Sci. 13, 809906. doi: 10.3389/fpls.2022.809906 35401626 PMC8987779

[B68] Islam F. Yasmeen T. Arif M. S. Ali S. Ali B. Hameed S.. (2016). Plant growth promoting bacteria confer salt tolerance in *Vigna radiata* by up-regulating antioxidant defense and biological soil fertility. Plant Growth Regul. 80, 23–36. doi: 10.1007/s10725-015-0142-y

[B69] Jaemsaeng R. Jantasuriyarat C. Thamchaipenet A. (2018). Molecular interaction of 1-aminocyclopropane-1-carboxylate deaminase (ACCD) producing endophytic streptomyces sp. GMKU 336 towards salt stress resistance of *Oryza sativa* l. cv. KDML105. Scien. Rep. 8 (1), 1–15. doi: 10.1038/s41598-018-19799-9 PMC5792428 29386629

[B70] Jalali F. Zafari D. Salari H. (2017). Volatile organic compounds of some Spp. increase growth and induce salt tolerance in. Fungal Ecol. 29, 67–75. doi: 10.1016/j.funeco.2017.06.007

[B71] Jaleel C. A. Sankar B. Sridharan R. Panneerselvam R. (2008). Soil salinity alters growth, chlorophyll content and secondary metabolite accumulation in *Catharanthus roseus* . Turk. J. Biol. 32 (2), 79–83.

[B72] Jalmi S. K. Sinha A. K. (2022). Ambiguities of PGPR induced plant signaling and stress management. Front. Microbiol. 13, 899563. doi: 10.3389/fmicb.2022.899563 35633696 PMC9136662

[B73] Ji J. Yuan D. Jin C. Wang G. Li X. Guan C. (2020). Enhancement of growth and salt tolerance of rice seedlings (*Oryza sativa* l.) by regulating ethylene production with a novel halo-tolerant PGPR strain glutamicibacter sp. YD01 containing ACC deaminase activity. Acta Physiol. Planta. 42 (4), 1–17. doi: 10.1007/s11738-020-3034-3

[B74] Jogawat A. (2019). “Osmolytes and their role in abiotic stress tolerance in plants,” in Molecular plant abiotic stress: Biology and biotechnology, vol. 2019 . Eds. Roychoudhury A. Tripathi D. (Hoboken, NJ, USA: John Wiley & Sons Ltd), 91–104.

[B75] Joshi E. B. Rajkumar S. (2019). Glucose and arabinose dependent mineral phosphate solubilization and its succinate mediated catabolite repression in rhizobium sp. RM and RS. J. Biosci. Bioengin. 128 (5), 551–557. doi: 10.1016/j.jbiosc.2019.04.020 31147219

[B76] Kamran M. Parveen A. Ahmar S. Malik Z. Hussain S. Chattha M. S.. (2019). An overview of hazardous impacts of soil salinity in crops, tolerance mechanisms and amelioration through selenium supplementation. Intern. J. Molec. Scien. 21 (1), 148. doi: 10.3390/ijms21010148 PMC6981449 31878296

[B77] Khan M. A. Asaf S. Khan A. L. Adhikari A. Jan R. Ali S.. (2019a). Halo-tolerant rhizobacterial strains mitigate the adverse effects of NaCl stress in soybean seedlings. Biomed. Res. Inter. 2019. doi: 10.1155/2019/9530963 PMC6925695 31886270

[B78] Khan M. A. Asaf S. Khan A. L. Ullah I. Ali S. Kang S. M.. (2019b). Alleviation of salt stress response in soybean plants with the endophytic bacterial isolate curtobacterium sp. SAK1. Ann. Microbiol. 69 (8), 797–808. doi: 10.1007/s13213-019-01470-x

[B79] Khan M. I. R. Asgher M. Khan N. A. (2014). Alleviation of salt induced photosynthesis and growth inhibition by salicylic acid involve glycine betaine and ethylene in mungbean (*Vigna radiata* l.). Plant Physiol. Biochem. 80, 67–74. doi: 10.1016/j.plaphy.2014.03.026 24727790

[B80] Khan M. A. Imran M. Shaffique S. Kwon E. H. Kang S. M. Kim S. H.. (2022). Mitigation of commercial food waste related salinity stress using halo-tolerant rhizobacteria in chinese cabbage plants. Horticul 8 (1), 49. doi: 10.3390/horticulturae8010049

[B81] Khan A. Zhao X. Q. Javed M. T. Khan K. S. Bano A. Shen R. F.. (2016). *Bacillus pumilus* enhances tolerance in rice (*Oryza sativa* l.) to combined stresses of NaCl and high boron due to limited uptake of na^+^ . Environ. Exp. Bot. 124, 120–129. doi: 10.1016/j.envexpbot.2015.12.011

[B82] Kim K. Jang Y. J. Lee S. M. Oh B. T. Chae J. C. Lee K. J. (2014). Alleviation of salt stress by enterobacter sp. EJ01 in tomato and *Arabidopsis* is accompanied by up-regulation of conserved salinity responsive factors in plants. Mol. Cells 37, 109–117. doi: 10.14348/molcells.2014.2239 24598995 PMC3935623

[B83] Kraegeloh A. Amendt B. Kunte H. J. (2005). Potassium transport in a halophilic member of the bacteria domain: identification and characterization of the k^+^ uptake systems TrkH and TrkI from *Halomonas elongata* DSM 2581T. J. Bacteriol. 187, 1036–1043. doi: 10.1128/JB.187.3.1036-1043.2005 15659681 PMC545715

[B84] Krishnamoorthy R. Choudhury A. Walitang D. I. Anandham R. Senthilkumar M. Sa T. (2022). Salt stress tolerance promoting proteins and metabolites under plant bacteria-salt stress tripartite interactions. Appl. Sci. 12 (6), 3126. doi: 10.3390/app12063126

[B85] Krulwich T. A. Sachs G. Padan E. (2011). Molecular aspects of bacterial pH sensing and homeostasis. Nat. Rev. Microbiol. 9 (5), 330–343. doi: 10.1038/nrmicro2549 21464825 PMC3247762

[B86] Kumar A. Singh S. Gaurav A. K. Srivastava S. Verma J. P. (2020). Plant growth promoting bacteria: biological tools for the mitigation of salinity stress in plants. Front. Microbiol. 11, 1216. doi: 10.3389/fmicb.2020.01216 32733391 PMC7358356

[B87] Kumar A. Verma J. P. (2018). Does plant microbe interaction confer stress tolerance in plants: A review? Microbiol. Res. 207, 41–52. doi: 10.1016/j.micres.2017.11.004 29458867

[B88] Kumawat K. C. Nagpal S. Sharma P. (2022a). Potential of plant growth promoting rhizobacteria plant interactions in mitigating salt stress for sustainable agriculture: A review. Pedosphere 32 (2), 223–245. doi: 10.1016/S1002-0160(21)60070-X

[B89] Kumawat K. C. Razdan N. Saharan K. (2022b). Rhizospheric microbiome: Bio-based emerging strategies for sustainable agriculture development and future perspectives. Microbiol. Res. 254, 126901. doi: 10.1016/j.micres.2021.126901 34700186

[B90] Kumawat K. C. Sharma P. Nagpal S. Gupta R. K. Sirari A. Nair R. M.. (2021). Dual microbial inoculation, a game changer? bacterial bio-stimulants with multifunctional growth promoting traits to mitigate salinity stress in spring mungbean. Front. Microbiol. 11, 600576. doi: 10.3389/fmicb.2020.600576 33584566 PMC7874087

[B91] Kumawat K. C. Singh I. Sharma P. Nagpal S. Gupta R. K. Sirari A. (2022c). Co-Inoculation of indigenous *Pseudomonas oryzihabitans* and bradyrhizobium sp. modulates growth, symbiotic efficacy, nutrient acquisition and grain yield in soybean. Pedosphere 32 (3), 438–451. doi: 10.1016/S1002-0160(21)60085-1

[B92] Lakshmi P. K. Usha C. (2022). Emerging technologies to understand plant microbe responses on climatic change. Plant Stress Mitigat., 451–468. doi: 10.1007/978-981-16-7759-5_21

[B93] Lipper L. Thornton P. Campbell B. M. Baedeker T. Braimoh A. Bwalya M. (2014). Climate smart agriculture for food security. Nat. Clim. Chang 4, 1068–1072. doi: 10.1038/nclimate2437

[B94] Lobell D. B. Schlenker W. Costa-Roberts J. (2011). Climate trends and global crop production since 1980. Scien 333, 616–620. doi: 10.1126/science.1204531 21551030

[B95] Lyu D. Backer R. Subramanian S. Smith D. L. (2020). Phyto-microbiome coordination signals hold potential for climate change resilient agriculture. Front. Plant Sci. 11, 634. doi: 10.3389/fpls.2020.00634 32523595 PMC7261841

[B96] Ma Y. Dias M. C. Freitas H. (2020). Drought and salinity stress responses and microbe induced tolerance in plants. Front. Plant Sci. 11, 591911. doi: 10.3389/fpls.2020.591911 33281852 PMC7691295

[B97] Mahmud-Ur-Rahman Naser I. B. Mahmud N. U. Sarker A. Hoque M. N. Islam T. A. (2022). Highly salt-tolerant bacterium *Brevibacterium sediminis* promotes the growth of rice (*Oryza sativa* l.) seedlings. Stresses 2, 275–289. doi: 10.3390/stresses2030020

[B98] Manh T. H. Garcia M. S. Vandecasteele M. Willems A. Luo D. Beirinckx S.. (2022). *Stenotrophomonas* sp. SRS1 promotes growth of *Arabidopsis* and tomato plants under salt stress conditions. Plant Soil 473 (1), 547–571. doi: 10.1007/s11104-022-05304-9

[B99] Marotkar S. Hirapure P. Paranjape S. Upadhye V. (2020). Crispr/Cas9 technology for crop improvement: A new weapon for Indian agricultural threats. Plant Cell Biotechnol. Molec. Biol. 21, 1–9.

[B100] Martynenko E. Arkhipova T. Safronova V. Seldimirova O. Galin I. Akhtyamova Z.. (2022). Effects of phyto-hormone producing rhizobacteria on casparian band formation, ion homeostasis and salt tolerance of durum wheat. Biomole 12, 230. doi: 10.3390/biom12020230 PMC8961637 35204731

[B101] Marulanda A. Azcon R. Chaumont F. Ruiz-Lozano J. M. Aroca R. (2010). Regulation of plasma membrane aquaporins by inoculation with a *Bacillus megaterium* strain in maize (*Zea mays* l.) plants under unstressed and salt stressed conditions. Planta 232, 533–543. doi: 10.1007/s00425-010-1196-8 20499084

[B102] Ma Y. Zhang W. Niu J. Ren Y. Zhang F. (2018). Hydrogen sulfide may function downstream of hydrogen peroxide in salt stress induced stomatal closure in *Vicia faba* . Funct. Plant Biol. 46 (2), 136–145. doi: 10.1071/FP18096 32172755

[B103] Mcnear D. H. (2013). The rhizosphere-roots, soil and everything in between. Nat. Educ. Knowl. 4, 1.

[B104] Minhas P. S. Bali A. Bhardwaj A. K. Singh A. Yadav R. K. (2021). Structural stability and hydraulic characteristics of soils irrigated for two decades with waters having residual alkalinity and its neutralization with gypsum and sulfuric acid. Agric. Water Manage. 244, 106609. doi: 10.1016/j.agwat.2020.106609

[B105] Mishra S. K. Khan M. H. Misra S. Dixit K. V. Khare P. Srivastava S.. (2017). Characterization of pseudomonas spp. and *Ochrobactrum* sp. isolated from volcanic soil. Antonie Van Leeuwenhoek 110 (2), 253–270. doi: 10.1007/s10482-016-0796-0 27853952

[B106] Moser R. Pertot I. Elad Y. Raffaelli R. (2008). Farmers’ attitudes toward the use of bio-control agents in IPM strawberry production in three countries. Biol. Contr. 47, 125–132. doi: 10.1016/j.biocontrol.2008.07.012

[B107] Mousavi S. S. Karami A. Saharkhiz M. J. Etemadi M. Ravanbakhsh M. (2022). Microbial amelioration of salinity stress in endangered accessions of Iranian licorice (*Glycyrrhiza glabra* l.). BMC Plant Biol. 22 (1), 1–17. doi: 10.1186/s12870-022-03703-9 35790900 PMC9254424

[B108] Mukhtar T. Shafiqur R. Smith D. Sultan T. Seleiman M. F. Alsadon A. A. (2020). Mitigation of heat stress in *Solanum lycopersicum* l. by ACC-deaminase and exo-polysaccharide producing *Bacillus cereus*: Effects on biochemical profiling. Sustainability 12, 2159. doi: 10.3390/su12062159

[B109] Munns R. Gilliham M. (2015). Salinity tolerance of crops-what is the cost? New Phytol. 208 (3), 668–673. doi: 10.1111/nph.13519 26108441

[B110] Mushtaq Z. (2020). PGPR: present role, mechanism of action and future prospects along bottlenecks in commercialization. EQA-Int. J. Environ. Q. 41, 9–15. doi: 10.6092/issn.2281-4485/11103

[B111] Naamala J. Smith D. L. (2020). Relevance of plant growth promoting microorganisms and their derived compounds, in the face of climate change. Agron 10, 1179. doi: 10.3390/agronomy10081179

[B112] Nagpal S. Sharma P. Kumawat K. C. (2019). Assessment of native single and dual inoculants of mesorhizobium sp. and endophytic rhizobacteria for plant growth promotion in chickpea. Agric. Res. J. 56 (4), 746–751.

[B113] Nagpal S. Sharma P. Sirari A. Gupta R. K. (2020). Co-Ordination of mesorhizobium sp. and endophytic bacteria as elicitor of bio-control against *Fusarium* wilt in chickpea. Europ. J. Plant Pathol. 158 (1), 143–161. doi: 10.1007/s10658-020-02062-1

[B114] Nascimento F. X. Hernández A. G. Glick B. R. Rossi M. J. (2020). Plant growth promoting activities and genomic analysis of the stress resistant *Bacillus megaterium* STB1, a bacterium of agricultural and biotechnological interest. Biotechnol. Rep. 25, e00406. doi: 10.1016/j.btre.2019.e00406 PMC6920507 31886139

[B115] Nautiyal C. S. Srivastava S. Chauhan P. S. Seem K. Mishra A. Sopory S. K. (2013). Plant growth-promoting bacteria *Bacillus amyloliquefaciens* NBRISN13 modulates gene expression profile of leaf and rhizosphere community in rice during salt stress. Plant Physiol. Biochem. 66, 1–9. doi: 10.1016/j.plaphy.2013.01.020 23454292

[B116] Niu S. Gao Y. Zi H. Liu Y. Liu X. Xiong X.. (2022). The osmolyte producing endophyte *Streptomyces albidoflavus OsiLf-2* induces drought and salt tolerance in rice *via* a multi-level mechanism. Crop J. 10 (2), 375–386. doi: 10.1016/j.cj.2021.06.008

[B117] Numan M. Bashir S. Khan Y. Mumtaz R. Shinwari Z. K. Khan A. L.. (2018). Plant growth promoting bacteria as an alternative strategy for salt tolerance in plants: A review. Microbiol. Res. 209, 21–32. doi: 10.1016/j.micres.2018.02.003 29580619

[B118] Ojuederie O. B. Babalola O. O. (2017). Microbial and plant assisted bio-remediation of heavy metal polluted environments: A review. Int. J. Environ. Res. Public Health 14, 1504. doi: 10.3390/ijerph14121504 29207531 PMC5750922

[B119] Okur B. Orçen N. (2020). “Soil salinization and climate change,” in Climate change and soil interactions (Elsevier), 331–350.

[B120] Ondrasek G. Rathod S. Manohara K. K. Gireesh C. Anantha M. S. Sakhare A. S.. (2022). Salt stress in plants and mitigation approaches. Plants 11 (6), 717. doi: 10.3390/plants11060717 35336599 PMC8950276

[B121] Pan T. Liu M. Kreslavski V. D. Zharmukhamedov S. K. Nie C. Yu M.. (2021). Non-stomatal limitation of photosynthesis by soil salinity. Crit. Rev. Environ. Sci. Technol. 51 (8), 791–825. doi: 10.1080/10643389.2020.1735231

[B122] Pan J. Xue X. Huang C. Peng F. Liao J. Ma S.. (2022). Salt tolerance strategies of *Nitraria tangutorum* bobr. and *Elaeagnus angustifolia* linn. determine the inoculation effects of microorganisms in saline soil conditions. Agron 12 (4), 913. doi: 10.3390/agronomy12040913

[B123] Parmar S. Gharat S. A. Tagirasa R. Chandra T. Behera L. Dash S. K.. (2020). Identification and expression analysis of miRNAs and elucidation of their role in salt tolerance in rice varieties susceptible and tolerant to salinity. PloS One 15 (4), e0230958. doi: 10.1371/journal.pone.0230958 32294092 PMC7159242

[B124] Patel D. Jha C. K. Tank N. Saraf M. (2012). Growth enhancement of chickpea in saline soils using plant growth promoting rhizobacteria. J. Plant Growth Regul. 31 (1), 53–62. doi: 10.1007/s00344-011-9219-7

[B125] Peng J. Ma J. Wei X. Zhang C. Jia N. Wang X.. (2021). Accumulation of beneficial bacteria in the rhizosphere of maize (*Zea mays* l.) grown in a saline soil in responding to a consortium of plant growth promoting rhizobacteria. Ann. Microbiol. 71, 40. doi: 10.1186/s13213-021-01650-8

[B126] Pereira S. I. Moreira H. Argyras K. Castro P. M. Marques A. P. (2016). Promotion of sunflower growth under saline water irrigation by the inoculation of beneficial microorganisms. Appl. Soil Ecol. 105, 36–47. doi: 10.1016/j.apsoil.2016.03.015

[B127] Prabhukarthikeyan S. R. Parameswaran C. Sawant S. B. Keerthana U. Yadav M. K. Raghu S.. (2022). Unraveling the molecular basis of *Bacillus megaterium* interactions in rice for plant growth promotion through proteomics and gene expression. J. Plant Growth Reg., 1–13. doi: 10.1007/s00344-022-10750-x

[B128] Prittesh P. Avnika P. Kinjal P. Jinal H. N. Sakthivel K. Amaresan N. (2020). Amelioration effect of salt tolerant plant growth promoting bacteria on growth and physiological properties of rice (*Oryza sativa*) under salt stressed conditions. Arch. Microbiol. 202 (9), 2419–2428. doi: 10.1007/s00203-020-01962-4 32591911

[B129] Qiang X. Ding J. Lin W. Li Q. Xu C. Zheng Q. (2019). Alleviation of the detrimental effect of water deficit on wheat (*Triticum aestivum* l.) growth by an indole acetic acid producing endophytic fungus. Plant Soil. 439, 373–391. doi: 10.1007/s11104-019-04028-7

[B130] Qiao Q. Wang F. Zhang J. Chen Y. Zhang C. Liu G. (2017). The variation in the rhizosphere microbiome of cotton with soil type, genotype and developmental stage. Sci. Rep. 7, 1–10. doi: 10.1038/s41598-017-04213-7 28638057 PMC5479781

[B131] Rabbani M. A. Maruyama K. Abe H. Khan M. A. Katsura K. Ito Y.. (2003). Monitoring expression profiles of rice genes under cold, drought and high-salinity stresses and abscisic acid application using cDNA microarray and RNA gel-blot analyses. Plant Physiol. 133 (4), 1755–1767. doi: 10.1104/pp.103.025742 14645724 PMC300730

[B132] Reinhold-Hurek B. Bunger W. Burbano C. S. Sabale M. Hurek T. (2015). Roots shaping their microbiome: global hotspots for microbial activity. Annu. Rev. Phytopathol. 53, 403–424. doi: 10.1146/annurev-phyto-082712-102342 26243728

[B133] Rodriguez-Navarro A. Rubio F. (2006). High affinity potassium and sodium transport systems in plants. J. Exp. Bot. 57 (5), 1149–1160. doi: 10.1093/jxb/erj068 16449373

[B134] Rojas-Tapias D. Moreno-Galvan A. Pardo-Diaz S. Obando M. Rivera D. Bonilla R. (2012). Effect of inoculation with plant growth promoting bacteria (PGPB) on amelioration of saline stress in maize (*Zea mays*). Appl. Soil Ecol. 61, 264–272. doi: 10.1016/j.apsoil.2012.01.006

[B135] Rolli E. de Zelicourt A. Alzubaidy H. Karampelias M. Parween S. Rayapuram N.. (2022). The lys motif receptor LYK4 mediates enterobacter sp. SA187 triggered salt tolerance in *Arabidopsis thaliana* . Environ. Microbiol. 24 (1), 223–239. doi: 10.1111/1462-2920.15839 34951090 PMC9304150

[B136] Roy Choudhury A. Roy S. K. Trivedi P. Choi J. Cho. K. Yun S. H.. (2022). Label free proteomics approach reveals candidate proteins in rice (*Oryza sativa* l.) important for ACC deaminase producing bacteria mediated tolerance against salt stress. Environ. Microbiol. 24 (8), 3612–3624. doi: 10.1111/1462-2920.15937 35191581

[B137] Safari D. Jamali F. Nooryazdan H. R. Bayat F. (2016). Screening fuorescent pseudomonads isolated from wheat rhizosphere for plant growth-promoting and salt tolerance properties. Biol. Forum. Int. J. 8, 35–42.

[B138] Safdar H. Amin A. Shafiq Y. Ali A. Yasin R. Shoukat A.. (2019). A review: Impact of salinity on plant growth. Nat. Sci. 17 (1), 34–40. doi: 10.7537/marsnsj170119.06

[B139] Sagar A. Sayyed R. Z. Ramteke P. W. Sharma S. Marraiki N. Elgorban A. M.. (2020). ACC deaminase and antioxidant enzymes producing halophilic enterobacter sp. PR14 promotes the growth of rice and millets under salinity stress. Physiol. Mol. Biol. Plants 26 (9), 1847–1854. doi: 10.1007/s12298-020-00852-9 32943820 PMC7468042

[B140] Sahab S. Suhani I. Srivastava V. Chauhan P. S. Singh R. P. Prasad V. (2021). Potential risk assessment of soil salinity to agro-ecosystem sustainability: Current status and management strategies. Sci. Total Environ. 764, 144164. doi: 10.1016/j.scitotenv.2020.144164 33385648

[B141] Saharan B. Nehra V. (2011). Plant growth promoting rhizobacteria: A critical review. Life Sci. Med. Res. 21, 30. doi: 10.4172/2157-7471.1000266

[B142] Sandhya V. Shrivastava M. Ali S. Z. Prasad V. S. S. K. (2017). Endophytes from maize with plant growth promotion and bio-control activity under drought stress. Russ. Agric. Sci. 43 (1), 22–34. doi: 10.3103/S1068367417010165

[B143] Sapre S. Gontia-Mishra I. Tiwari S. (2018). *Klebsiella* sp. confers enhanced tolerance to salinity and plant growth promotion in oat seedlings (*Avena sativa*). Microbiol. Res. 206, 25–32. doi: 10.1016/j.micres.2017.09.009 29146257

[B144] Sapre S. Gontia-Mishra I. Tiwari S. (2021). Plant growth promoting rhizobacteria ameliorates salinity stress in pea (*Pisum sativum*). J. Plant Growth Regul. 41, 647–656. doi: 10.1007/s00344-021-10329-y

[B145] Sarkar J. Chakraborty B. Chakraborty U. (2018). Plant growth promoting rhizobacteria protects wheat plants against temperature stress through antioxidant signaling and reducing chloroplast and membrane injury. J. Plant Growth Regul. 37, 1396–1412. doi: 10.1007/s00344-018-9789-8

[B146] Selvakumar G. Kim K. Hu S. Sa T. (2014). “Effect of salinity on plants and the role of arbuscular mycorrhizal fungi and plant growth promoting rhizobacteria in alleviation of salt stress,” in Physiological mechanisms and adaptation strategies in plants under changing environment (New York: Springer), 115–144.

[B147] Sen S. Chandrasekhar C. N. (2014). Effect of PGPR on growth promotion of rice (*Oryza sativa* l.) under salt stress. Asian J. Plant Sci. 4 (5), 62–67.

[B148] Shafi A. Zahoor I. Mushtaq U. (2019). “Proline accumulation and oxidative stress: Diverse roles and mechanism of tolerance and adaptation under salinity stress,” in Salt stress, microbes, and plant interactions: Mechanisms and molecular approaches (Singapore: Springer), 269–300.

[B149] Shahid M. A. Sarkhosh A. Khan N. Balal R. M. Ali S. Rossi L.. (2020). Insights into the physiological and bio-chemical impacts of salt stress on plant growth and development. Agron 10 (7), 938. doi: 10.3390/agronomy10070938

[B150] Shahid S. A. Zaman M. Heng L. (2018). “Soil salinity: Historical perspectives and a world overview of the problem,” in Guideline for salinity assessment, mitigation and adaptation using nuclear and related techniques (Cham: Springer), 43–53.

[B151] Shah A. Nazari M. Antar M. Msimbira L. A. Naamala J. Lyu D.. (2021). PGPR in agriculture: A sustainable approach to increasing climate change resilience. Front. Sustain. Food Syst. 5. doi: 10.3389/fsufs.2021.667546

[B152] Sharif M. Ghorbanli M. Ebrahimzadeh H. (2007). Improved growth of salinity stressed soybean after inoculation with salt pre-treated mycorrhizal fungi. J. Plant Physiol. 164, 1144–1151. doi: 10.1016/j.jplph.2006.06.016 16919369

[B153] Sharma D. K. (2014). “Sustainable technologies for crop production under salt affected soil in India,” in Proceedings of 3rd international salinity forum, session, vol. 2. UC Riverside Convention Center, Riverside, CA, USA.

[B154] Sharma D. K. Chaudhari S. K. (2012). Agronomic research in salt affected soils of India: an overview. Indian J. Agron. 57, 175–185.

[B155] Sheteiwy M. S. Elgawad H. A. Xiong Y. C. Macovei A. Brestic M. Skalicky M.. (2021). Inoculation with bacillus amyloliquefaciens and mycorrhiza confers tolerance to drought stress and improve seed yield and quality of soybean plant. Physiol. Plantarum 172 (4), 2153–2169. doi: 10.1111/ppl.13454 33964177

[B156] Shivanand P. Mugeraya G. (2011). Halophilic bacteria and their compatible solutes osmo-regulation and potential applications. Curr. Sci. 100, 1516–1521. Available at: https://www.jstor.org/stable/24076671

[B157] Shrivastava P. Kumar R. (2015). Soil salinity: A serious environmental issue and plant growth promoting bacteria as one of the tools for its alleviation. Saud. J. Biol. Sci. 22 (2), 123–131. doi: 10.1016/j.sjbs.2014.12.001 PMC4336437 25737642

[B158] Shukla P. S. Agarwal P. K. Jha B. (2012). Improved salinity tolerance of *Arachis hypogaea* (L.) by the interaction of halo-tolerant plant growth promoting rhizobacteria. J. Plant Growth Regul. 31 (2), 195–206. doi: 10.1007/s00344-011-9231-y

[B159] Singh G. (2018). Climate change and sustainable management of salinity in agriculture. Res. Med. Eng. Sci. 6 (2), 1–7. doi: 10.31031/RMES.2018.06.000635

[B160] Singh R. P. Jha P. N. (2016). The multifarious PGPR serratia marcescens CDP-13 augments induced systemic resistance and enhanced salinity tolerance of wheat (*Triticum aestivum* l.). PloS One 11 (6), e0155026. doi: 10.1371/journal.pone.0155026 27322827 PMC4913913

[B161] Singh R. P. Ma Y. Shadan A. (2022a). Perspective of ACC deaminase producing bacteria in stress agriculture. J. Biotechnol. 352, 36–46. doi: 10.1016/j.jbiotec.2022.05.002 35597331

[B162] Singh R. P. Pandey D. M. Jha P. N. Ma Y. (2022b). ACC deaminase producing rhizobacterium *Enterobacter cloacae* ZNP-4 enhance abiotic stress tolerance in wheat plant. PloS One 17 (5), e0267127. doi: 10.1371/journal.pone.0267127 35522667 PMC9075627

[B163] Song Y. Zheng H. Sui Y. Li S. Wu F. Sun X.. (2022). SbWRKY55 regulates sorghum response to saline environment by its dual role in abscisic acid signaling. Theor. Appl. Gene. 135 (8), 2609–2625. doi: 10.1007/s00122-022-04130-y 35841419

[B164] Srivastava P. Wu Q. S. Giri B. (2019). “Salinity: An overview,” in Microorganism in saline environments: Strategies and functions. Soil Biology (Switzerland: Springer Nature), 56, 3–18. doi: 10.1007/978-3-030-18975-4_1

[B165] Stolte J. Tesfai M. Oygarden L. Kværnø S. Keizer J. Verheijen F.. (2015). “Soil threats in Europe: status, methods, drivers and effects on ecosystem services,” in A review report, deliverable 2.1 of the RECARE project, vol. 27607. (Luxembourg: Office for Official Publications of the European Community), 69–78.

[B166] Suarez C. Cardinale M. Ratering S. Stefens D. Jung S. Montoya A. M. Z.. (2015). Plant growth promoting effects of *Hartmannibacter diazotrophicus* on summer barley (*Hordeum vulgare* l.) under salt stress. Appl. Soil Ecol. 95, 23–30. doi: 10.1016/j.apsoil.2015.04.017

[B167] Subramanyam K. Laing G. Van Damme E. J. (2019). Sodium selenate treatment using a combination of seed priming and foliar spray alleviates salinity stress in rice. Front. Plant Sci. 10, 116. doi: 10.3389/fpls.2019.00116 30804974 PMC6378292

[B168] Sultana R. Zuan A. T. Yusop M. R. Saud H. M. (2020). Characterization of salt tolerant plant growth promoting rhizobacteria and the effect on growth and yield of saline affected rice. PloS One 15 (9), e0238537. doi: 10.1371/journal.pone.0238537 32886707 PMC7473536

[B169] Tabassum B. Khan A. Tariq M. Ramzan M. Khan M. S. I. Shahid N. (2017). Bottlenecks in commercialization and future prospects of PGPR. Appl. Soil Ecol. 121, 102–117. doi: 10.1016/j.apsoil.2017.09.030

[B170] Tewari S. Arora N. K. (2016). Fluorescent pseudomonas sp. PF17 as an efficient plant growth regulator and bio-control agent for sunflower crop under saline conditions. Symbiosis 68 (1), 99–108. doi: 10.1007/s13199-016-0389-8

[B171] Tiwari S. Nutan K. K. Deshmukh R. Sarsu F. Gupta K. J. Singh A. K.. (2022). Seedling stage salinity tolerance in rice: decoding the role of transcription factors. Physiol. Plant 174 (2), e13685. doi: 10.1111/ppl.13685 35419814

[B172] Tripathi A. Chacon O. Lata Singla-Pareek S. K. Sopory S. Sanan-Mishra N. (2018). Mapping the microRNA expression profiles in glyoxalase over-expressing salinity tolerant rice. Curr. Geno. 19 (1), 21–35. doi: 10.2174/1389202918666170228134530 PMC5817874 29491730

[B173] Tufail M. A. Touceda-González M. Pertot I. Ehlers R. U. (2021). *Gluconacetobacter diazotrophicus* Pal5 enhances plant robustness status under the combination of moderate drought and low nitrogen stress in *Zea mays* l. Microorganisms 9, 870. doi: 10.3390/microorganisms9040870 33920684 PMC8073419

[B174] Ullah S. Bano A. Ullah A. Shahid M. A. Khan N. (2022). A comparative study of plant growth promoting rhizobacteria (PGPR) and sowing methods on nutrient availability in wheat and rhizosphere soil under salinity stress. Rhizosph 23, 100571. doi: 10.1016/j.rhisph.2022.100571

[B175] Vimal S. R. Patel V. K. Singh J. S. (2019). Plant growth promoting *Curtobacterium albidum* strain SRV4: an agriculturally important microbe to alleviate salinity stress in paddy plants. Ecol. Ind. 105, 553–562. doi: 10.1016/j.ecolind.2018.05.014

[B176] Wang D. Cao W. Zhang F. Li Z. Xu S. Wu X. (2022a). A review of deep learning in multiscale agricultural sensing. Remote Sens. 14 (3), 559. doi: 10.3390/rs14030559

[B177] Wang G. Li B. Peng D. Zhao H. Lu M. Zhang L.. (2022b). Combined application of H_2_S and a plant growth promoting strain JIL321 regulates photosynthetic efficacy, soil enzyme activity and growth promotion in rice under salt stress. Microbiol. Res. 256, 126943. doi: 10.1016/j.micres.2021.126943 34953293

[B178] Xu H. Gao J. Portieles R. Du L. Gao X. Borras-Hidalgo O. (2022). Endophytic bacterium *Bacillus aryabhattai* induces novel transcriptomic changes to stimulate plant growth. PloS One 17 (8), e0272500. doi: 10.1371/journal.pone.0272500 35921359 PMC9348713

[B179] Yadav R. Chakraborty S. Ramakrishna W. (2022). Wheat grain proteomic and protein metabolite interactions analyses provide insights into plant growth promoting bacteria arbuscular mycorrhizal fungi wheat interactions. Plant Cell Rep. 41, 1417–1437. doi: 10.1007/s00299-022-02866-x 35396966

[B180] Yan K. He W. Bian L. Zhang Z. Tang X. An M.. (2020). Salt adaptability in a halophytic soybean (*Glycine soja*) involves photo-systems coordination. BMC Plant Biol. 20 (1), 1–13. doi: 10.1186/s12870-020-02371-x 32276592 PMC7149873

[B181] Yan J. Smith M. D. Glick B. R. Liang Y. (2014). Effects of ACC deaminase containing rhizobacteria on plant growth and expression of toc GTPases in tomato (*Solanum lycopersicum*) under salt stress. Botany 92 (11), 775–781. doi: 10.1139/cjb-2014-0038

[B182] Yasmin H. Naeem S. Bakhtawar M. Jabeen Z. Nosheen A. Naz R. (2020). Halo-tolerant rhizobacteria *Pseudomonas pseudoalcaligenes* and *Bacillus subtilis* mediate systemic tolerance in hydroponically grown soybean (*Glycine max* l.) against salinity stress. PloS One 15, e0231348. doi: 10.1371/journal.pone.0231348 32298338 PMC7162512

[B183] Yun P. Xu L. Wang S. S. Shabala L. Shabala S. Zhang W. Y. (2018). Piriformospora indica improves salinity stress tolerance *in zea mays* l. plants by regulating na^+^ and k^+^ loading in root and allocating k^+^ in shoot. Plant Growth Reg. 86, 323–331. doi: 10.1007/s10725-018-0431-3

[B184] Zarea M. J. Hajinia S. Karimi N. Goltapeh E. M. Rejali F. Varma A. (2012). Effect of *Piriformospora indica* and *Azospirillum* strains from saline or non-saline soil on mitigation of the effects of NaCl. Soil Biol. Biochem. 45, 139–146. doi: 10.1016/j.soilbio.2011.11.006

[B185] Zawoznik M. S. Ameneiros M. Benavides M. P. Vázquez S. Groppa M. D. (2011). Response to saline stress and aquaporin expression in *Azospirillum* inoculated barley seedlings. Appl. Microbiol. Biotechnol. 90 (4), 1389–1397. doi: 10.1007/s00253-011-3162-1 21365472

[B186] Zhang S. Fan C. Wang Y. Xia Y. Xiao W. Cui X. (2018). Salt tolerant and plant growth promoting bacteria isolated from high yield paddy soil. Can. J. Microbiol. 64, 968–978. doi: 10.1139/cjm-2017-0571 30148967

[B187] Zhao Y. Zhang F. Mickan B. Wang D. Wang W. (2022). Physiological, proteomic and metabolomic analysis provide insights into bacillus sp. mediated salt tolerance in wheat. Plant Cell Rep. 41 (1), 95–118. doi: 10.1007/s00299-021-02788-0 34546426

[B188] Zhao C. Zhang H. Song C. Zhu J. K. Shabala S. (2020). Mechanisms of plant responses and adaptation to soil salinity. Innovation 1 (1), 100017. doi: 10.1016/j.xinn.2020.100017 34557705 PMC8454569

[B189] Zilaie M. N. Arani A. M. Etesami H. Dinarvand M. (2022). Halo-tolerant rhizobacteria enhance the tolerance of the desert halophyte *Nitraria schoberi* to salinity and dust pollution by improving its physiological and nutritional status. Appl. Soil Ecol. 179, 104578. doi: 10.1016/j.apsoil.2022.104578

[B190] Zizkova E. Dobrev P. I. Muhovski Y. Hosek P. Hoyerova K. Haisel D.. (2015). Tomato (*Solanum lycopersicum* l.) SlIPT3 and SlIPT4 isopentenyl transferases mediate salt stress response in tomato. BMC Plant Biol. 15, 85. doi: 10.1186/s12870-015-0415-7 25888402 PMC4404076

